# Supplementary material for: Development of a digital, self-guided return-to-work toolkit for stroke survivors and employers using intervention mapping
Source: PLOS Digit Health. 2025 Aug 6;4(8):e0000971. doi: 10.1371/journal.pdig.0000971 (PMC12327610; doi:10.1371/journal.pdig.0000971)
Supplement: S2 Table — (DOCX) [file pdig.0000971.s002.docx]

**S2. Adapted TIDieR (Template for Intervention Description and Replication) Checklist.**

| **Item** | **Description** | **Location in Manuscript** | **Other Sources** |
| --- | --- | --- | --- |
| **1. Brief Name** | Provide a concise name or phrase describing the intervention. | p.1, p.7 |  |
| **2. Rationale** | Explain the theory, objectives, or reasoning that underpin the intervention. | pp.4-7, pp.14-20 |  |
| **3. Materials** | List any tools, handouts, or other resources used in the intervention and where they can be accessed. | pp.24-25 |  |
| **4. Procedures** | Describe the steps, techniques, or processes involved in delivering the intervention. | p.22 | Supporting file S10 |
| **5. Provider** | Identify the type of person delivering the intervention, including their expertise or training. | N/A (self-guided, p.24) |  |
| **6. Mode of Delivery** | Indicate how the intervention was delivered (e.g., face-to-face, online), and whether individually or in a group. | p.22, p.24 |  |
| **7. Location** | Describe the setting where the intervention was delivered, and any relevant contextual features. | N/A |  |
| **8. Schedule** | Report how often the intervention was delivered, its duration, and over what time period. | p.24 |  |
| **9. Tailoring** | If the intervention was designed to be personalised or adapted, explain how and why. | p.24 |  |
| **10. Modifications** | Describe any changes to the intervention during the study and the reasons for them. | pp.23-24 |  |
| **11. Planned Fidelity** | Outline how you intended to monitor or maintain fidelity to the intervention plan. | N/A |  |
| **12. Actual Fidelity** | Describe how closely the delivered intervention matched what was planned. | N/A |  |

Note: This checklist has been adapted from the original TIDieR checklist published in BMJ 2014;348:g1687. The structure and intent of the items have been retained, while the accompanying guidance has been reworded to avoid any copyright issues.

Citation: Hoffmann TC, Glasziou PP, Boutron I, et al. Better reporting of interventions: Template for Intervention Description and Replication (TIDieR) checklist and guide. BMJ 2014;348:g1687.
