# Supplementary material for: Development of a digital, self-guided return-to-work toolkit for stroke survivors and employers using intervention mapping
Source: PLOS Digit Health. 2025 Aug 6;4(8):e0000971. doi: 10.1371/journal.pdig.0000971 (PMC12327610; doi:10.1371/journal.pdig.0000971)
Supplement: S1 Text — (DOCX) [file pdig.0000971.s003.docx]

**S1. Detailed description of methods per intervention-mapping step.**

- 1. **Step 1: Logic model of the problem**

The desired outcome of the first intervention-mapping (IM) step is a ‘logic model of the problem,’ depicting the factors that influence or cause the problem the intervention will be targeting [1]. The suggested model is created through consultation with stakeholders (i.e., a planning group) and a needs assessment.

- - 1. **Establishing stakeholder input to assist with intervention planning**
    2. **Setting up an expert advisory group**

Expert advisory groups provide technical guidance and support in a particular area, through group meetings or correspondence [2]. KC consulted with the research team to make a list of potential group members. In July 2022, potential members from the list were invited via email to attend three group meetings over a two-year period to advise on project delivery. One-to-one support would be requested as needed on specific topics. The invite email was also distributed to members of the Council for Work and Health. It was planned that the advisory group would give feedback on the design of data collection for the study (i.e., needs assessment and workshops), the logic model of change, and intervention prototypes.

The IM approach suggests setting up a planning group of community members, intervention users, and potential implementers to ensure the project focus and outcomes are relevant [1]. Given that employers are a ‘hard-to-reach’ population [3], it was anticipated that few members of the advisory group may be able to offer input from the employer perspective. To maximise opportunity for employer stakeholder input, three workshops were planned with additional employers. It was planned that the workshops would involve decisions on priority areas and goals of the intervention (workshop 1), brainstorming of ideas for intervention design and content (workshop 2), and gaining feedback on the intervention name, logo, and intervention plan (workshop 3).

- - 1. **Workshops with employers**

Employers were recruited through convenience and snowball sampling. The study was advertised between February 2023-January 2024, through X, LinkedIn, the Different Strokes website, gatekeepers (e.g., expert advisory group members, charities, professional membership organisations), and face-to-face at business networking events in the East and West Midlands, United Kingdom.

Eligible participants were required to be aged 18 years or older; work in an occupational role involving staff responsibility; and proficient in use of English language. Potential participants contacted KC via email to receive the relevant participant information. Participation involved taking part in any of three, two-hour workshops via Microsoft Teams. Verbal informed consent was taken one-to-one in a brief Microsoft Teams meeting prior to the workshop/s, and documented on a Microsoft Word form, signed on the participant’s behalf. Completed verbal consent forms (.docx) were sent to participants, and copies saved securely within Microsoft Teams. Workshops were recorded and transcribed using Microsoft Teams; recordings (.mp4) and anonymised transcriptions (.docx) were saved on Microsoft Teams.

- 1. **Needs assessment**

A mixed-methods study was conducted to explore employers’ needs for providing return-to-work (RTW) support to stroke survivor employees [4]. The first part consisted of a qualitative systematic review, exploring employers’ views on factors influencing their support [5]. Further exploration was undertaken through interviews with employers (n=7). An online quantitative survey of employers (n=50) investigated knowledge of stroke and the RTW process, and perceived competency (i.e., employers’ belief in their ability to learn and execute skills) for carrying out supportive actions for RTW. Findings from the review, interviews, and survey were synthesised using the triangulation approach [6]. The synthesised findings were then placed into a logic model of the problem (Supporting file S5).

The first annual meeting with the expert advisory group took place in September 2022, after the systematic review (but before the survey and interviews). Group members were presented with the systematic review findings and asked for advice on recruitment and data collection in the survey and interviews. Initial ideas on intervention scope/content were also requested.

- 1. **Workshop 1. Selecting priority areas and intervention goals**

During workshop 1 in August 2023, KC presented the needs assessment findings to participants. Based on this, participants were then tasked with selecting priority areas for the intervention to focus on. Problematic behaviours or conditions relating to employers, other stakeholders, e.g., stroke survivors, and environment were summarised in a diagram. Participants were invited to discuss the findings as a group, and make any further comments. They were then asked to vote on the top three behaviours or environment conditions the intervention should focus on, using a multiple-choice poll. Next, they ranked their importance using a ranking poll. During task 2, they were asked to consider the three priority areas selected, and discuss and decide on the determinants to be targeted. In line with IM terminology, a determinant was defined as anything influencing people’s behaviours, such as knowledge or environmental conditions. In two breakout rooms, participants were asked to construct one goal for one priority area selected, using the guiding question, ‘*What* will change for *whom*, by *how much*, over what *time*?’ Input from workshop 1 was used to refine the logic model of the problem (Supporting file S6).

1. **Step 2: Intervention outcomes and objectives (logic model of change)**

The desired outcome of the second IM step is a ‘logic model of change,’ depicting pathways of intervention effects [1]. Using the logic model of the problem, behavioural outcomes for employers and stroke survivors were listed. KC then constructed lists of performance objectives. Searches were made for research evidence to identify further possible determinants (in addition to those listed in the logic model of the problem). Following on from this, lists of determinants were constructed and mapped onto domains of the Theoretical Domains Framework (TDF) [7, 8]. The IM approach recommends use of relevant theory to identify determinants and inform selection of intervention methods. The TDF was selected for this purpose because it is designed to support assessment of determinants of behaviours [8]. TDF constructs were also relevant to employers and organisational settings, e.g., social/professional role and identity, environmental context and resources.

Behavioural outcomes, performance objectives, and determinants were then inputted into a matrix to define change objectives (Supporting file S7). KC constructed and presented the logic model of change to the research team and expert advisory group, and made changes in line with feedback.

1. **Step 3: Intervention design**

Step 3 of the IM approach involves matching theory- and evidence-based behaviour change methods to the determinants selected in step 2; and selection of appropriate practical applications [1].

- 1. **Selection of theory-based behaviour change methods**

To select theory-based behaviour change methods, KC created a separate table for each determinant, and included within the table relevant behaviour change methods and parameters (i.e., conditions needed for success), and ideas for practical applications (Supporting file S8). This information was organised according to the change and performance objectives, and intervention step they related to. Behaviour change methods, parameters, and ideas for practical applications were obtained from the IM approach’s taxonomy of behaviour change methods [9]. KC also searched PubMed for evidence-based behaviour change methods for the determinants. However, no relevant literature was identified. This is not unheard of at this stage of the IM approach [1]. Articles describing intervention development do not always report logic models of change, nor include detail on intervention methods.

- 1. **Selection of practical applications**

KC searched on PubMed and Google Scholar for eHealth workplace interventions for employers and/or employees with injuries or health conditions. In a table, the eHealth intervention findings relating to practical applications (i.e., their usability, effectiveness, and acceptability) were mapped onto relevant determinants and performance objectives from the matrices of change (Supporting file S9).

To further inform selection of practical applications, workshop 2 was carried out in October 2023. Participants were shown a summary diagram of change and performance objectives for employers and stroke survivors, organised into a five-step process. They were then asked for ideas on content, i.e., ‘tools,’ that could help employers and stroke survivors carry out the objectives shown in the diagram. For inspirational purposes, they were presented with ideas from the first expert advisory group meeting, and visual examples of RTW tools from online resources, e.g., a RTW decision tree, written prompts to aid disclosure to employers, and a workplace adjustment passport. During task 2, they were asked to suggest ideas on intervention design and testing. Questions referred to the intervention’s design features and implementation, e.g., form (website, PDF, e-learning package), funding for its development, storage, and testing, and plans for user access. During the first two advisory group meetings, members were also asked for ideas on practical applications and the design and implementation of the intervention.

1. **Step 4: Intervention production**

Step 4 of the IM approach involves organising, condensing, and producing the list of practical applications gained during step 3. All performance objectives, determinants, intervention methods, practical applications, and parameters (conditions) for success were tabulated, organised per intervention step. Two of these tables were created; one for employers and one for stroke survivors.

A five-step plan was created, showing the sequence, scope and content of each intervention step, organised per user group (employers and stroke survivors) (Supporting file 10). The key focus of each step and corresponding key messages were included within the plan, alongside suggested tasks for employers and stroke survivors. General ideas from stakeholders and eHealth intervention evidence (see step 3) regarding intervention design and delivery were included within the plan.

In February 2024, workshop 3 took place. An update on the project was given. During task 1, participants were shown a selection of potential intervention logos and asked to indicate the one they liked best via a voting poll. During task 2, they were asked to give feedback on the proposed intervention plan. For each intervention step, they were shown a summary of the key messages and a list of the tools to accompany those messages. As part of this task, they were also presented with PDFs containing ‘tools’ to facilitate suggested actions/behaviours (i.e., performance objectives) per step, and asked for feedback. KC took notes during the workshop and referred to the workshop transcript to make suggested changes to the intervention messages and tools. The intervention prototype was created using Xerte [10], a content authoring platform that enables development of interactive learning materials.

As part of step 4, the IM approach also recommends pretesting intervention materials and messages with intended users before final production [1]. Pretesting is carried out prior to pilot-testing and is considered crucial for ensuring the intervention is implementable, and its materials comprehensible and appealing. It was planned that the expert advisory group would pre-test and provide this initial feedback (Table 2). To ensure input from as wide a range of potential users as possible, additional stroke survivors and employers were invited to join the expert advisory group, i.e., through KC presenting at a local stroke research partnership group and emailing contacts in the NHS. Three additional stroke survivors and three employers agreed to join the advisory group.

KC then emailed all advisory group members with an invitation to provide feedback on the intervention prototypes. Stroke survivors were invited to review the stroke survivor version of the intervention, and employers invited to review the employer version. They were also given access to each other’s versions if they wished to review those also. Other group members, e.g., occupational therapists could choose the version/s they wished to review. Group members who responded were asked to complete a series of simple tasks, e.g., navigate to a page containing a video and play it. Two weeks later, they were asked to attend one of three separate Microsoft Team meetings for 1) stroke survivors, 2) employers and 3) other group members), to answer open-ended questions regarding the prototype’s acceptability, ease of use/learnability, accessibility and inclusivity, perceived usefulness, and issues affecting use (technical or environmental). These questions were based on constructs from the Technology Acceptance Model [11, 12], System Usability Scale [13], and International Classification of Functioning, Disability and Health [14] (Supporting file S4). Group members were also asked how they thought potential users should be made aware of and gain access to the intervention. Those unable to attend meetings responded to the questions via email.

Deductive framework analysis [15] was conducted on meeting transcripts and email responses, guided by the question constructs for coding, using NVivo version 12 [16] and Microsoft Excel (version 16.65).

1. Bartholomew-Eldridge L, Markham C, Ruiter R, Fernández M, Kok G, Parcel. Planning health promotion programs: An intervention mapping approach. 4th ed. San Francisco, CA: Jossey-Bass; 2016.

2. World Health Organization. Expert advisory panels and committees. n.d.

3. Coole C, Nouri F, Narayanasamy M, Baker P, Khan S, Drummond A. Engaging workplace representatives in research: What recruitment strategies work best? Occup Med. 2018;68(4):282-5.

4. Craven K, Kettlewell J, De Dios Pérez B, Powers K, Holmes J, Radford KA. What do employers need when supporting stroke survivors to return to work?: a mixed-methods study. Top Stroke Rehabil. 2024:1-13.

5. Craven K, De Dios Pérez B, Holmes J, Fisher R, Radford KA. Factors influencing employers’ support for employees with acquired brain injuries or mental illness to return to- and stay in work: A qualitative systematic review. Work. 2024;79(1):93-121.

6. Farmer T, Robinson K, Elliott SJ, Eyles J. Developing and Implementing a Triangulation Protocol for Qualitative Health Research. Qual Health Res. 2006;16(3):377-94.

7. Atkins L, Francis J, Islam R, O’Connor D, Patey A, Ivers N, et al. A guide to using the Theoretical Domains Framework of behaviour change to investigate implementation problems. Implementation science : IS. 2017;12(1):77-.

8. Cane J, O'Connor D, Michie S. Validation of the theoretical domains framework for use in behaviour change and implementation research. Implementation science 2012;7(1):37-.

9. Kok G, Gottlieb NH, Peters G-JY, Mullen PD, Parcel GS, Ruiter RAC, et al. A taxonomy of behaviour change methods: an Intervention Mapping approach. Health Psychol Rev. 2016;10(3):297-312.

10. The Xerte Project: University of Nottingham; [Available from: <https://www.nottingham.ac.uk/xerte/>.

11. Davis F. Perceived Usefulness, Perceived Ease of Use, and User Acceptance of Information Technology. 1989.

12. Davis FD, Bagozzi RP, Warshaw PR. User Acceptance of Computer Technology: A Comparison of Two Theoretical Models. Management science. 1989;35(8):982-1003.

13. Brooke J. SUS: A ‘quick and dirty’ usability scale. In: P. W. Jordan BT, B. A. Weerdmeester, & A. L. McClelland, editor. Usability evaluation in industry. London: Taylor and Francis; 1996. p. 189-94.

14. World Health Organization. International Classification of Functioning, Disability, and Health (ICF). Geneva: World Health Organization; 2001.

15. Gale NK, Heath G, Cameron E, Rashid S, Redwood S. Using the framework method for the analysis of qualitative data in multi-disciplinary health research. BMC Med Res Methodol. 2013;13(1):117-.

16. QSR International Pty Ltd. NVivo qualitative data analysis software (version 12). 2018.
