# Supplementary material for: Development of a digital, self-guided return-to-work toolkit for stroke survivors and employers using intervention mapping
Source: PLOS Digit Health. 2025 Aug 6;4(8):e0000971. doi: 10.1371/journal.pdig.0000971 (PMC12327610; doi:10.1371/journal.pdig.0000971)
Supplement: S3 Table — (DOCX) [file pdig.0000971.s004.docx]

**S3. Pre-test feedback questions.**

**Key:**

TAM= Technology Acceptance Model [1, 2]

SUS= System Usability Scale [3]

ICF= International Classification of Functioning, Disability and Health [4]

**Initial questions:**

1. Devices used to access TTEAM?
2. Prototype/s accessed?
   1. Stroke survivor version
   2. Employer version
   3. Both

**Questions for discussion:**

| **Theoretical construct** | **Question** |
| --- | --- |
| Perceived ease of use/learnability (TAM/SUS) | Anything that was **helpful** when accessing and navigating TTEAM?  Anything that **made it difficult** to access and navigate TTEAM? |
| Accessibility and inclusivity (ICF) | **If you have an impairment/disability,** was there anything you:   - liked about TTEAM in relation to your needs? - did **not** like about TTEAM in relation to your needs? |
| Perceived usefulness (TAM) | Anything about TTEAM that was **useful** for learning about stroke and returning to work? |
| **General questions:**  Could anything be changed to improve TTEAM?  How should people be made aware of and access TTEAM?  Is there anything else you would like to say (or ask)?  (Note: the following questions were asked if they had already not been addressed by the previous responses) | |
| Functional relevance (ICF) | How was the content of TTEAM relevant and applicable for stroke survivors and employers navigating the return to work process? (Encourage linking content with own real-life experiences) |
| Technical issues (SUS) | Can you describe any technical issues experienced while using TTEAM?     - Were there any ways of overcoming these? |
| Environmental factors and context (ICF) | Did anything about your environment affect your experience using TTEAM today?     - E.g., Noise, lighting, technology setup? |
| User satisfaction (SUS) | Overall, how satisfied are you with TTEAM?     - Would they suggest any changes? |
| Acceptance and willingness to use/recommend (TAM) | Would you be use TTEAM again or recommend it to others?    What factors influenced their answer? |

**References**

1. Davis F. Perceived Usefulness, Perceived Ease of Use, and User Acceptance of Information Technology. 1989.

2. Davis FD, Bagozzi RP, Warshaw PR. User Acceptance of Computer Technology: A Comparison of Two Theoretical Models. Management science. 1989;35(8):982-1003.

3. Brooke J. SUS: A ‘quick and dirty’ usability scale. In: P. W. Jordan BT, B. A. Weerdmeester, & A. L. McClelland, editor. Usability evaluation in industry. London: Taylor and Francis; 1996. p. 189-94.

4. World Health Organization. International Classification of Functioning, Disability, and Health (ICF). Geneva: World Health Organization; 2001.
