# Supplementary figures and images for: Development of a digital, self-guided return-to-work toolkit for stroke survivors and employers using intervention mapping
Source: PLOS Digit Health. 2025 Aug 6;4(8):e0000971. doi: 10.1371/journal.pdig.0000971 (PMC12327610; doi:10.1371/journal.pdig.0000971)

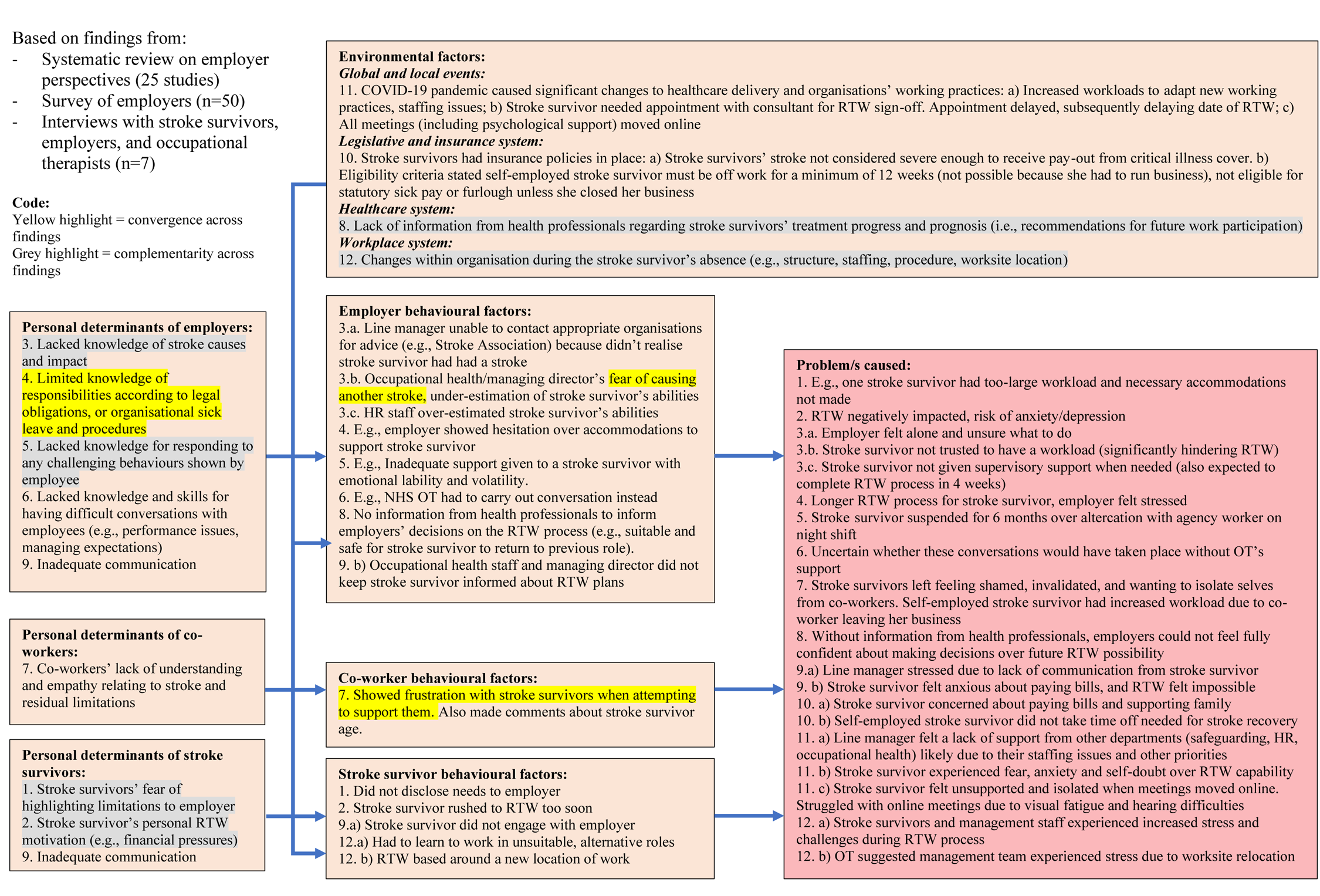

Supplement: S1 Fig — (TIF) [file pdig.0000971.s005.tif]

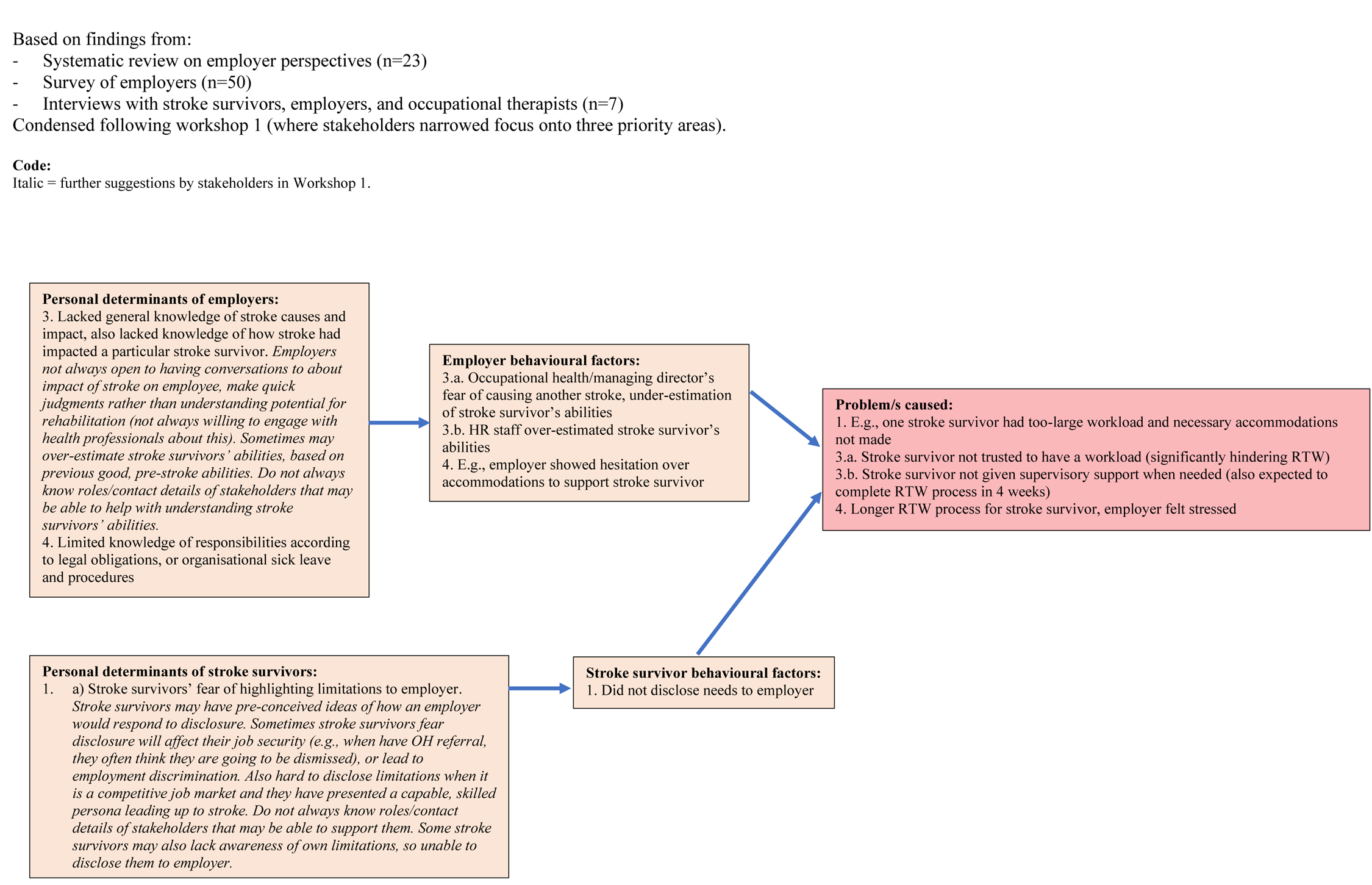

Supplement: S2 Fig — (ZIP) [file pdig.0000971.s006.zip › S2_Fig/S2_Fig]
