# Supplementary material for: Development of a digital, self-guided return-to-work toolkit for stroke survivors and employers using intervention mapping
Source: PLOS Digit Health. 2025 Aug 6;4(8):e0000971. doi: 10.1371/journal.pdig.0000971 (PMC12327610; doi:10.1371/journal.pdig.0000971)
Supplement: S4 Table — (DOCX) [file pdig.0000971.s007.docx]

**S4. Matrices of change.**

|  | **Behaviour 1: Stroke survivors disclose essential needs to employer** | | | | |
| --- | --- | --- | --- | --- | --- |
|  | **Determinants (mapped onto TDF domains)** | | | | |
| **Performance objectives**  **(Stroke survivors)** | Stroke survivor may believe work caused their stroke, or worry about future impact of work on their health (Knowledge, Beliefs about Consequences, Emotion)* | Stroke survivor may not know if they are ready to start preparing for return to work  (Intentions, Beliefs about Capabilities, Knowledge) | Stroke survivors don’t always know who/how to contact people that can help them understand their capabilities, limitations, and needs (Knowledge) | Stroke survivors not aware of their capabilities and limitations (Knowledge and Behavioural Regulation) | Stroke survivors fear highlighting limitations to employer (Emotion and Beliefs about Consequences) |
| PO.1. Reflect on- and communicate readiness (to/with employer) to start planning for returning to work | *K.1.a./BaCo.1.a.Obtain contact details and liaise with stakeholders (e.g., consultant, GP, health psychologist, occupational therapist) who could educate them on the cause/s of their stroke, and advise on work participation*** | *I.1.a. Reflect on the benefits of returning to work (e.g., maintain financial income, maintain social relationships, sense of purpose and routine, etc)*  *I.1.b. Reflect on aspects of the environment that could facilitate return to work (e.g., physical aspects like work environment, social support available [e.g., supportive co-workers], etc)*  *BaCa.1./K.1.b. Recognise the following:*  *- A gradual approach to planning and returning to work is needed*  *- Their capabilities may not reach their pre-stroke level again (and that is okay)*  *- A trial and error approach will be needed*  *-They will need to participate in action planning at regular timepoints before, during, and after the return-to-work date* |  |  |  |
|  |  |  |  |  |  |
| PO.2. Appraise capabilities and limitations in relation to working role |  |  | *K.2.a. Find out roles and contact details of relevant stakeholders (e.g., occupational therapist, occupational health physician) who could help with appraisal* | *K.2.b. Contact stakeholders (including family and other stroke survivors***, employers or co-workers [i.e., ‘buddies’], if needed) for support with appraisal of limitations and needs, and communication of needs to employer.*  *K.2.c. Appraise abilities needed for working role*  *K.2.d. Appraise and compare current functional abilities to abilities needed for working role*  *K.2.e. Identify strengths and limitations for working role* |  |
| PO.3. Identify what is needed to enable work participation |  |  |  | *K.3. Consider strengths and limitations, and identify needs (e.g., adjustments) for work participation* |  |
| PO.4. Consider what needs are essential for the employer to know for provision of support |  |  |  | *K.4. Decide which identified needs are essential for enabling or maintaining work participation* |  |
| PO.5. Clearly communicate essential needs to employer |  |  |  |  | *BaCo.5.a./E.5.a. Recognise that disclosing limitations could lead to more realistic expectations and better provision of support from employer (e.g., through needs being met)*  *BaCo.5.b./E.5.b. Recognise that open communication regarding limitations could help maintain relationships with employer and co-workers***** |
|  |  |  |  |  | *K.5. Liaise with relevant stakeholders to plan how best to communicate essential needs to employer (e.g., face-to-face, in writing with copies for both)* |
| PO.6. Repeat PO. 2-5, regularly review needs with employer on ongoing basis, as agreed (e.g., monthly basis) |  |  |  | *K.6. Recognise that limitations can change*  *BR.6. Decide how/when to record and review limitations and needs with employer*  *BR.6.c. Regularly review and record limitations and needs with employer* |  |

*Illness perceptions, as defined by: Leventhal H, Phillips LA, Burns E. The Common-Sense Model of Self-Regulation: a dynamic framework for understanding illness self-management. 2016.

**By gaining detailed and clear information and instruction to inform illness representations and action plans, this should help adherence to self-management of these in the return to work context.

***Sometimes family and employers have unrealistic expectations of stroke survivor. Brannigan C, Galvin R, Walsh ME, Loughnane C, Morrissey EJ, Macey C, et al. Barriers and facilitators associated with return to work after stroke: a qualitative meta-synthesis. Disability and rehabilitation. 2017;39(3):211–22.

**** Based on recommendations from: Li JY, Lee Y. To Disclose or Not? Understanding Employees’ Uncertainty and Behavior Regarding Health Disclosure in the Workplace: A Modified Socioecological Approach. International journal of business communication (Thousand Oaks, Calif). 2023;60(1):173–201.

|  | **Behaviour 2: Employers increase and maintain understanding of stroke survivors’ abilities** | | | | | **Behaviour 3: Employers provide reasonable adjustments for stroke survivors when needed** | | | **Behaviours 2 and 3** | |
| --- | --- | --- | --- | --- | --- | --- | --- | --- | --- | --- |
|  | **Determinants (mapped onto TDF domains)** | | | | | | | | | |
| **Performance objectives**  **(Employers)** | Employers don’t always know who can help them with understanding stroke survivor’s abilities (Knowledge) | Employers not always willing to engage in communications about stroke survivors’ abilities (e.g., due to perceptions that internal HR or OH have everything in hand*) (Social/Professional Role and Identity) | Employers lack general knowledge of stroke causes and impact (Knowledge) | Employers lack knowledge of specific impact of stroke on stroke survivor (Knowledge) | Employers over-estimate stroke survivor abilities based on good pre-stroke abilities (Knowledge) | Employers have limited knowledge of responsibilities relating to the return-to-work process, e.g., according to legal obligations, or organisational sick leave and procedures (Knowledge) | Employers anxious about potential impact on co-workers from stroke survivor’s sickness absence or return to work (Emotions, Beliefs about Consequences) | Employers’ concern about cost of reasonable adjustments* (Emotions, Environmental context and resources) | Employers may not have confidence or competence for carrying out reasonable adjustments and RTW process actions (Skills, Beliefs about Capabilities) | Employers have pre-conceived beliefs about stroke and possibility of RTW (e.g., interview ppt 01) (Knowledge, Beliefs about Consequences) |
| PO.1. Contact stroke survivor and jointly agree communication schedule | *K.1.a. Recognise the potential benefits to liaising with a stroke survivor (and family) about their abilities*  *(e.g., for understanding their abilities and providing more tailored support)* |  |  |  |  | *K.1.b. Recognise the importance and potential benefits of early, regular communication with employees post-stroke, as recommended by the Stroke Association*, Acas**, CIPD***, and SOM* |  |  | *S1.a./BaCa.1.a. Reflect on their confidence and skills for contacting and communicating with stroke survivor*  *S.1.b./BaCa.1.b. Identify and attend relevant training, review educational materials, or consult relevant individuals to learn how to communicate with stroke survivors* | *K.1.c. Recognise that stroke affects individuals differently*  *BaCo.2. Recognise that with the right support, stroke survivors can sometimes successfully return to- and stay in work* |
| PO.2. Recognise limited general knowledge of stroke |  |  | *K.2. Evaluate general knowledge about stroke causes and impact* |  |  |  |  |  |  |  |
| PO.3. Recognise limited knowledge of specific impact of stroke on stroke survivor |  |  |  | *K.3. Evaluate knowledge about specific impact of stroke on stroke survivor* | *BaCa.3. Recognise that stroke survivor may have residual limitations affecting work abilities (some of which may be invisible)* |  |  |  |  |  |
| PO.4. Recognise how stroke survivor’s return to work may impact wider team (e.g., who does tasks, how they’re done, co-workers’ feelings) |  |  |  |  |  |  | *E.4.a./BaCo.4.a. Discuss with co-workers potential impact of stroke survivor’s sickness absence and their return to work (e.g., on their work tasks and wellbeing)* |  |  |  |
| PO.5. Recognise limited understanding of responsibilities during return-to-work process |  |  |  |  |  | *K.5. Evaluate knowledge of responsibilities relating to the return-to-work process (e.g., making reasonable adjustments, informing them about resources available)* |  |  |  |  |
| PO.6. Recognise limited understanding of organisational resources available to stroke survivor employee |  |  |  |  |  | *K.6. Evaluate knowledge of internal and external resources available to support stroke survivor employee* |  |  |  |  |
| PO.7. Recognise need for support with identifying and organising reasonable adjustments for stroke survivor, that are affordable and don’t put any employees’ health and safety at risk. |  | *SPRI.7.a. Recognise that other stakeholders (e.g., occupational therapists) are specially trained to recommend reasonable adjustments in line with stroke survivors’ abilities*  *SPRI.7.b. Recognise the potential benefits to liaising with a stroke survivor (and family) about their abilities (e.g., for understanding their abilities and providing more tailored support)* |  |  | *BaCa.7.a. Recognise that stroke survivor may need reasonable adjustments to facilitate work participation* | *K.7. Recognise that provision of reasonable adjustments is their responsibility according to the Equality Act 2010* | *E.7.a./BaCo.7. Reflect on concerns regarding potential impact of stroke survivor’s return on employees’ health and safety* | *E.7.b./ECR.7. Recognise that reasonable adjustments are an investment to aid retention of the stroke survivor* | *S.7./BaCa.7.b. Reflect on confidence and skills for identifying and organising reasonable adjustments* |  |
| PO.8. Identify appropriate stakeholders and sources for support with 1-7 (with consent from stroke survivor if needed) | *See the following:*  *SPRI.8.b.*  *K.8.a.*  *K.8.b.*  *BaC.8.a.* | *SPRI.8.a. Recognise importance of knowing specific impact of stroke on stroke survivor (i.e., for own role in providing support)*  *SPRI.8.b. Recognise that external stakeholders may know more about a stroke survivor’s abilities than the organisation* | *K.8.a. Recognise that there are stakeholders, educational sources and/or training that can improve general knowledge of stroke* | *K.8.b. Recognise that there are stakeholders, educational sources and/or training that can improve knowledge of specific impacts of stroke (e.g., fatigue)* | *BaCa.8.a. Recognise that some stakeholders (e.g., occupational therapists) are specially trained to assess stroke survivors’ post-stroke work abilities* | *K.8.c. Recognise that there are stakeholders, informational sources, and/or training that can improve knowledge of employer responsibilities relating to the return-to-work process* | *E.8.a./BaCo.8. Recognise that relevant stakeholders and organisations can advise on measures to protect health and safety of employees, and manage impact of the stroke survivor’s return (e.g., strategies to maintain productivity and maintain wellbeing)* | *E.8.b./ECR.8.a. Recognise there may be stakeholders ( internal and external to organisation) who may be able to allocate resources for reasonable adjustments*  *E.8.c./ECR.8.b. Recognise that relevant stakeholders can advise on making adjustments in cost-effective way* | *S.8./Baca.8.b. Recognise that there are stakeholders, informational sources, and/or training that can educate them on how to carry out reasonable adjustments and RTW process actions (e.g., plan phased return, conduct work trial)* |  |
| PO.9. Liaise with relevant stakeholders (including stroke survivor) or organisations, review information, or attend training | *K.9.a. Obtain contact details of stakeholders who can help with understanding stroke survivor’s abilities (with consent from stroke survivor)* | *See previous objectives in this column* | *K.9.b. Obtain contact details of stakeholders, collate educational information, and/or organise training to improve general knowledge of stroke* | *K.9.c. Obtain contact details of stakeholders, collate educational information and/or organise training to improve knowledge of specific impacts of stroke (e.g., fatigue)* | *See K.9.a.* | *K.9.d.Obtain contact details of stakeholders, collate information and/or organise training to improve knowledge of employer responsibilities relating to the return-to-work process* | *E.9.a./BaCo.9. Obtain contact details of stakeholders and organisations who can advise on measures to protect health and safety of employees* | *ECR.9. Obtain contact details of stakeholders who can advise on organisational resources and/or cost-effectiveness of reasonable adjustments* | *S.9./BaCa.9. Recognise potential benefits of learning RTW process skills and practicing them to improve confidence*  *K.9.e. Obtain contact details of stakeholders, collate educational information, and/or organise training to improve skills and confidence for providing reasonable adjustments and carrying out RTW process actions* |  |
| PO.10. Regularly review stroke survivor’s needs (with stroke survivor) on ongoing basis as agreed (e.g., monthly basis), repeat PO.9. |  |  |  |  | *K.10. Recognise that a stroke survivor’s limitations can change*  *BaCa.10.b. Decide with stroke survivor how/when to record and review work abilities of stroke survivor* |  | *E.10.a./BaCo.a. Recognise that requirements for measures to protect employee health and wellbeing can change*  *E.10.b./BaCo.10.b. Decide with relevant stakeholders how/when to record and review protective measures for employees’ health and safety*  *E.10.c./BaCo.10.c. Regularly record and review protective measures for employees’ health and safety*  *E.10.d./BaCo.10.d. Analyse whether changes to protective measures for employees’ health and safety are needed* | *E.10.a/ECR.10.a. Recognise that resources (e.g., funds, staff availability) for reasonable adjustments can change*  *E.10.b./ECR.10.b. Decide with relevant stakeholders how/when to record and review resources for reasonable adjustments needed*  *E.10.c./ECR.10.c. Regularly record and review resources for reasonable adjustments needed*  *E.10.d./ECR.10.d. Analyse whether changes in resources affect opportunity to provide reasonable adjustments* | *S.10.a./BaCa.10.a. Regularly reflect on skills and confidence relating to RTW process actions (including making reasonable adjustments)*  *S.10.b./BaCa.10.b. Evaluate whether further training or educational support (including practice and feedback) is needed to improve or maintain skills and confidence for RTW process actions* |  |

* https://www.stroke.org.uk/sites/default/files/publications/a_complete_guide_to_stroke_for_employers_1.pdf

** <https://www.acas.org.uk/keeping-in-touch-during-absence>

*** <https://www.cipd.org/uk/knowledge/guides/managing-return-to-work-after-long-term-absence/>

**** <https://www.som.org.uk/sites/som.org.uk/files/Occupational_Health_The_Value_Proposition_March_2022.pdf>
