# Supplementary material for: Development of a digital, self-guided return-to-work toolkit for stroke survivors and employers using intervention mapping
Source: PLOS Digit Health. 2025 Aug 6;4(8):e0000971. doi: 10.1371/journal.pdig.0000971 (PMC12327610; doi:10.1371/journal.pdig.0000971)
Supplement: S5 Table — (DOCX) [file pdig.0000971.s008.docx]

**S5. Selection of theory-based behaviour change methods and applications.**

1. This table demonstrates which tables in the intervention mapping taxonomy [1] were used for each determinant (to aid selection of appropriate theory-based behaviour change methods).

| **Determinants coded using Theoretical Domains Framework (TDF) [2]** | **Translation for people unfamiliar with TDF** | **Related categories of theoretical change methods (according to intervention-mapping guidance)** |
| --- | --- | --- |
| Behavioural regulation | Self-monitoring, then planning and changing behaviours if needed | - Basic methods at the individual level - Methods to influence skills, capability, and self-efficacy and to overcome barriers |
| Skills | Skills |  |
| Beliefs about capabilities  (Employer beliefs about their capabilities overlapped with their skills, so included in same table) | Confidence relating to own capabilities |  |
| Beliefs about consequences  Emotion  (Put together in same table because they overlapped) | What people think will happen as a result of a behaviour in a given context/scenario | - Basic methods at the individual level - Methods to change attitudes, beliefs, and outcome expectations |
| Intentions | Conscious decision to perform a behaviour | - Basic methods at the individual level - Methods to change attitudes, beliefs, and outcome expectations - Methods to influence skills, capability, and self-efficacy and to overcome barriers |
| Knowledge | Knowledge | - Basic methods at the individual level - Methods to increase knowledge |
| Social/Professional role and identity | How people perceive their social/professional role | - Basic methods at the individual level - Methods for changing social influence |

1. These are the tables matching theory-based intervention methods and practical strategies (i.e., applications) to the determinants and change/performance objectives (using the intervention mapping taxonomy).

| **Determinant: Behavioural regulation**  **(Stroke survivors not aware of their capabilities and limitations)** | | | | | | |
| --- | --- | --- | --- | --- | --- | --- |
| **Steps in intervention process** | **Performance objectives for stroke survivor** | **Change objectives** | **Methods** | **Definition** | **Parameters** | **Application (i.e., linked toolkit content)** |
| 5 | PO.6. Repeat PO. 2-5, regularly review needs with employer on ongoing basis, as agreed (e.g., monthly basis) | Decide how/when to record and review limitations and needs with employer | **Participation** | Assuring engagement of participants in problem-solving, decision making, and change activities | All participants need to be accepted by convenor as having high influence. Participants need motivation and skills | E.g., information on role of each participant (employer/stroke survivor) in process, ask each participant to sign this document at the end to signify that they have read and understood their role in regular reviews? Document to be kept in stroke survivor employee record? |
|  |  |  | **Public commitment** | Stimulating pledging to perform the desired behaviour, and announcing that decision to others | Most effective when announced to other/s, may include contracting |  |
|  |  | Regularly review and record limitations and needs with employer | **Implementation intentions** | Prompting if-then plans, linking situational cues with responses effective in attaining desired outcomes | Existing positive intention | Reminder notifications for regular reviews (could be as simple as setting up calendar alerts). Brief message in each alert saying purpose of meeting, and who should be involved (stroke survivor and employer) |
|  |  |  | **Goal setting** | Prompting planning what the person will do, including a definition of goal-directed behaviours that result in the target behaviour | Commitment to the goal; goals that are difficult but available within the individual’s skill level | Reasonable adjustments passport. Look at what was done since previous review (i.e., were goals achieved? Review of previous reasonable adjustments, and changes needed for future. Could include section at end for goals until next review period (and who should complete the related actions) |
|  |  |  | **Active learning** | Encouraging learning from goal-driven and activity-based experience | Time, information, and skills |  |
|  |  |  | **Self-monitoring of behaviour** | Prompting the person to keep a record of specified behaviour/s | Monitoring must be of the specific behaviour. The data must be interpreted and used. The reward must be reinforcing to the individual |  |

| **Determinant: Skills/Beliefs about Capabilities**  **(Employers may not have confidence or competence for carrying out reasonable adjustments and RTW process actions)** | | | | | | |
| --- | --- | --- | --- | --- | --- | --- |
| **Steps in intervention process** | **Performance objectives for employer** | **Change objectives** | **Method** | **Definition** | **Parameters** | **Application (i.e., linked toolkit content)** |
| 1 | PO.1. Contact stroke survivor and jointly agree communication schedule | Reflect on their confidence and skills for contacting and communicating with stroke survivor | **Active learning** | Encouraging learning from goal-driven and activity-based experience | Time, information and skills  Will need to be brief exercise. | Reflective prompts/quiz |
|  |  | Identify and attend relevant training, review educational materials, or consult relevant individuals to learn how to communicate with stroke survivors | **Goal setting** | Prompting planning what the person will do, including a definition of goal-directed behaviours that result in the target behaviour/s | Commitment to the goal; goals that are difficult but available within the individual’s skill level | E.g., Ask employers to make a list of learning/training activities they will do? |
|  |  |  | **Self-monitoring of behaviour** | Prompting the person to keep a record of specified behaviour/s (e.g., training completed) | The monitoring must be of the specific behaviour/s. Data must be interpreted and used. Reward must be reinforcing to the individual | E.g., put it in performance review/training record, looks good to their organisation |
|  |  |  | **Planning coping responses** | Prompting participants to list potential barriers (e.g., not knowing what to do if a stroke survivor becomes upset) | Identification of high-risk situations and practice of coping response (e.g., what to say) | Examples to prompt |
|  |  |  | **Active learning (also links to planning coping responses and goal setting above)** | Encouraging learning from goal-driven and activity-based experience | Time, information and skills | Signposting in toolkit to potential training, educational materials or people who may be able to educate/practice communication.  Ask to fill in a communication schedule with stroke survivor as a task? |
| 4 | PO.7. Recognise need for support with identifying and organising reasonable adjustments for stroke survivors, that are affordable and don’t put any employees’ health and safety at risk. | Reflect on confidence and skills for identifying and organising reasonable adjustments | **Enactive mastery experience** | Provide increasingly challenging tasks with feedback to serve as indicators of capability | Requires willingness to accept feedback | E.g., After receiving information about reasonable adjustments, perform simulated scenario exercises, selecting appropriate reasonable adjustments for different stroke survivor cases |
|  |  |  | **Active learning (also overlaps with enactive mastery experience)** | Encouraging learning from goal-driven and activity-based experience | Time, information and skills  Will need to be brief exercise. | Reflective prompts/quiz |
| 5 | PO.8. Regularly review stroke survivor’s needs (with stroke survivor) on ongoing basis as agreed (e.g., monthly basis), repeat PO. 5-7 and identification of- and liaison with relevant stakeholders. | Regularly reflect on skills and confidence relating to RTW process actions (including making reasonable adjustments) | **Public commitment** | Stimulating pledging, promising, or engaging oneself to perform the desired behaviour and announcing that decision to others | Needs to be a public announcement may include contracting | E.g., As part of review contract with stroke survivor and their role competencies, employer promises to ensure they maintain/obtain the skills needed to carry out RTW process actions |
|  |  |  | **Self-monitoring of behaviour** | Prompting the person to keep a record of specified behaviour/s | Data must be interpreted and used. Reward must be reinforcing to individual. | Record their actions on RA passport/record of actions (like clinical notes). Data can be used to inform performance review? |
|  |  | Evaluate whether further training or educational support (including practice and feedback) is needed to improve or maintain skills and confidence for RTW process actions | **Persuasive communication** | Guiding individuals and environmental agents toward the adoption of an idea, attitude, or action by using argument or other means | Messages need to be relevant and not too discrepant from individuals’ beliefs. Can be stimulated by surprise and repetition, will include arguments | Information on the importance of employers evaluating whether they have educational or training needs throughout RTW process. Can be emphasised in role play videos showing examples of “good” and “bad” employers, or when stroke survivors talk about their RTW experiences |
|  |  |  | **Active learning** | Encouraging learning from goal-driven and activity-based experience | Time, information and skills  Will need to be brief exercise. | Could ask employers to write a list of educational and training needs at regular timepoints (e.g., after each review with stroke survivors) |
| All steps | Relevant to all POs. Identify appropriate stakeholders and sources for support with (with consent from stroke survivor if needed). | Recognise that there are stakeholders, informational sources, and/or training that can educate them on how to carry out reasonable adjustments and RTW process actions (e.g., plan phased return, conduct work trial) | **Persuasive communication** | Guiding individuals and environmental agents toward the adoption of an idea, attitude, or action by using argument or other means | Messages need to be relevant and not too discrepant from individuals’ beliefs. Can be stimulated by surprise and repetition, will include arguments | Information on the roles and skills of relevant stakeholders who may be available to support (e.g., occupational health/therapy, human resources, stroke charities, training courses, Health and Safety Executive, trade union, etc).  Examples of stakeholders’ input in role play videos? |
|  | Relevant to all POs. Liaise with relevant stakeholders (including stroke survivor) or organisations, review information, or attend training. | Recognise potential benefits of learning RTW process skills and practicing them to improve confidence | **Persuasive communication** | Guiding individuals and environmental agents toward the adoption of an idea, attitude, or action by using argument or other means | Messages need to be relevant and not too discrepant from individuals’ beliefs. Can be stimulated by surprise and repetition, will include arguments | Information on the potential benefits of employers learning and maintaining RTW process skills. Can be mentioned in relation to job competencies, role play videos showing examples of “good” and “bad” employers, or when stroke survivors talk about their RTW experiences |
|  |  |  | **Participation** | Assuring engagement of participants in problem-solving, decision making, and change activities | All participants need to be accepted by convenor as having high influence. Participants need motivation and skills | See above – emphasise benefits of liaising with relevant stakeholders (including stroke survivor) |

| **Determinant: Beliefs about capabilities**  **(Stroke survivor may not know if they are ready to start preparing for return to work)** | | | | | | |
| --- | --- | --- | --- | --- | --- | --- |
| **Steps in intervention process** | **Performance objectives for stroke survivor** | **Change objectives** | **Methods** | **Definition** | **Parameters** | **Application (i.e., linked toolkit content)** |
| 1 | PO.1. Reflect on- and communicate readiness (to/with employer) to start planning for returning to work | Recognise the following:  - A gradual approach to planning and returning to work is needed  - Their capabilities may not reach their pre-stroke level again (and that is okay)  - A trial and error approach will be needed  -They will need to participate in action planning at regular timepoints before, during, and after the return-to-work date | **Modelling** | Providing an appropriate model; being reinforced for their desired action | Attention, remembrance, self-efficacy, and skills; reinforcement of model; coping model instead of mastery model | E.g., Show how different stroke survivors have managed to do all of these things (emphasising not always at pre-stroke level) and successfully prepared for RTW:  E.g., Give  real-life examples: Results from stroke VR/RTW studies, videos of stroke survivors telling of their experience relating to this, and written stories with image (option to use either) |
|  |  |  | **Persuasive communication**  **(This could also be classed as verbal persuasion)** | Guiding individuals and environmental agents toward the adoption of an idea, attitude, or action by using argument or other means | Messages need to be relevant and not too discrepant from individuals’ beliefs. Can be stimulated by surprise and repetition, will include arguments |  |
|  |  |  | **Verbal persuasion** | Using messages that suggest that the participant possesses certain capabilities | Credible source | See above |
|  |  |  | **Active learning** | Encouraging learning from goal-driven and activity-based experience | Time, information and skills  Will need to be brief exercise. | Checklist to tick whether they feel willing and able to undertake that aspect of the RTW process? E.g., Gradual approach if needed, trial-and-error approach, etc. |

| **Determinant: Beliefs about Consequences/Emotions**  **(Stroke survivor may believe work caused their stroke, or worry about future impact of work on their health)**  **(Stroke survivors fear highlighting limitations to employer)** | | | | | | |
| --- | --- | --- | --- | --- | --- | --- |
| **Steps in intervention process** | **Performance objectives for stroke survivor** | **Change objectives** | **Methods** | **Definition of method** | **Parameters** | **Application (i.e., linked toolkit content)** |
| 1 | PO.1. Reflect on- and communicate readiness (to/with employer) to start planning for returning to work | Obtain contact details and liaise with stakeholders (e.g., consultant, GP, health psychologist, occupational therapist) who could educate them on the cause/s of their stroke, and advise on work participation***** | **Persuasive communication** | Guiding individuals and environmental agents toward the adoption of an idea, attitude, or action by using argument or other means | Messages need to be relevant and not too discrepant from individuals’ beliefs. Can be stimulated by surprise and repetition, will include arguments | Message stating how relevant stakeholders could advise on cause/s of stroke and advise on work participation. Information on causes of most strokes (%). Include health psychologists as one of stakeholders mentioned (e.g. in relation to managing distressing thoughts like “work caused my stroke” – acceptance and commitment therapy). |
|  |  |  | **Active learning** | Encouraging learning from goal-driven and activity-based experience | Time, information and skills | Task could be to identify and make list of stakeholders to contact to understand more about cause/s of stroke and advice for work participation (if possible). Then to populate template based on their responses. |
|  |  |  | **Modelling** | Providing an appropriate model; being reinforced for their desired action | Attention, remembrance, self-efficacy, and skills; reinforcement of model; coping model instead of mastery model | E.g., Show how different stroke survivors with different residual limitations and occupational roles have managed to RTW, with no negative impact on health.  E.g., Give  real-life examples – videos, written stories with images, categorised by types of residual limitations? Give as many examples with different roles/ages/ethnicities/industries/organisation sizes as possible |
|  |  |  | **Cultural similarity** | Using characteristics of the target group in source, message, and channel | Using surface characteristics of the target group enhances receptivity. Using socio-cultural characteristics leads to a more positive reception of the message | E.g., see above – messages given by stroke survivors, etc |
| 3 | PO.5. Clearly communicate essential needs to employer | Recognise that disclosing limitations could lead to more realistic expectations and better provision of support from employer (e.g., through needs being met) | **Arguments** | Using a set of one or more meaningful premises and a conclusion | For central processing of arguments they need to be new to the message receiver. | Potential benefits for and against disclosure. Argue that if done in the right way, it can be beneficial, e.g., essential needs disclosed, selective on sharing with people who will be supportive. Inform them of their rights – e.g., once they disclose their needs, the employer is legally obligated to provide reasonable adjustments. Could cite research evidence, e.g., qualitative, to argue for this. What happened when people did and did not disclose. |
|  |  |  | **Modelling** | Providing an appropriate model; being reinforced for the desired action | Attention, remembrance, self-efficacy and skills, reinforcement of model, identification with model, coping model instead of mastery model | E.g., Videos of stroke survivors saying what happened when they disclosed their limitations, videos showing what happened when they didn’t. Alternative is to write two scenarios (and/or play them as infographic film) showing this from qualitative research findings (needs assessment interviews). |
|  |  |  | **Environmental re-evaluation** | Encouraging realising the negative impact of the unhealthy behaviour (i.e., non-disclosure), and the positive impact of the healthy behaviour (i.e., disclosure) | Stimulation of both cognitive and affective appraisal to improve appraisal and empathy skills |  |
|  |  | Recognise that open communication regarding limitations could help maintain relationships with employer and co-workers | **Environmental re-evaluation** | Encouraging realising the negative impact of the unhealthy behaviour (i.e., non-disclosure), and the positive impact of the healthy behaviour (i.e., disclosure) | Stimulation of both cognitive and affective appraisal to improve appraisal and empathy skills | See above  Could include brief quotes/testimonials from stroke survivors re. impact of open communication |
|  |  |  | **Arguments** | Using a set of one or more meaningful premises and a conclusion | For central processing of arguments they need to be new to the message receiver. | Potential benefits for and against disclosure, in relation to relationships with co-workers and employer. Argue that if done in the right way, it can be beneficial, social support obtained – vital for RTW. If not done, people may not be understanding or offer support where needed, relationships could diminish due to lack of understanding. If shared with the wrong people, could perhaps be detrimental then too. Tips on knowing the types of people to share with?  Could cite research evidence, e.g., qualitative, to argue for this. Could also refer to videos where relevant. |
| **Determinant: Beliefs about Consequences/Emotions**  **(Employers anxious about potential impact on co-workers from stroke survivor’s sickness absence or return to work)**  **(Employers have pre-conceived beliefs about stroke and possibility of RTW)** | | | | | | |
| **Steps in intervention process** | **Performance objectives for employer** | **Change objectives** | **Methods** | **Definition of method** | **Parameters** | **Application (i.e., linked toolkit content)** |
| 1 | PO.1. Contact stroke survivor and jointly agree communication schedule | Recognise that with the right support, stroke survivors can sometimes successfully return to- and stay in work | **Persuasive communication** | Guiding agents towards adoption of an idea, attitude, or action by using argument or other means | Messages should be relevant, not too different from individual’s beliefs. Can be stimulated by surprise and repetition | Message: with the right support, stroke survivors can RTW. Give examples from research literature.  E.g., Videos of successful RTW stories – stroke survivors talking about their experiences and how employers helped them (or didn’t). |
|  |  |  | **Arguments** | Using a set of one or more meaningful premises and a conclusion | For central processing of arguments they need to be new to the message receiver. |  |
| 4 | PO.6. Recognise how stroke survivor’s return to work may impact wider team (e.g., who does tasks, how they’re done, co-workers’ feelings) | Discuss with co-workers potential impact of stroke survivor’s sickness absence and their return to work (e.g., on their work tasks and wellbeing) | **Modelling** | Providing an appropriate model; being reinforced for the desired action | Attention, remembrance, self-efficacy and skills, reinforcement of model, identification with model, coping model instead of mastery model | E.g., Video showing employer carrying out discussion in way that respects stroke survivor’s wishes re. disclosure |
|  | PO.7. Recognise need for support with identifying and organising reasonable adjustments for stroke survivors, that are affordable and don’t put any employees’ health and safety at risk. | Reflect on concerns regarding potential impact of stroke survivor’s return on employees’ health and safety | **Shifting perspective** | Encouraging taking perspective of the other | Initiation from the perspective of the learner; needs imaginary competence | E.g., Imaginary exercise. Employer imagines day working as stroke survivor in work role/environment (with their disabilities). Notes down health and safety issues that may affect stroke survivor and/or co-workers.  Another idea: Reflective prompts on things to consider, link to overview of stakeholder roles, e.g., who to contact for support with risk assessments |
|  |  |  | **Active learning** | Encouraging learning from goal-driven and activity-based experience | Time, information and skills  Will need to be brief exercise. |  |
| 5 | PO.8. Regularly review stroke survivor’s needs (with stroke survivor) on ongoing basis as agreed (e.g., monthly basis), repeat PO. 5-7 | Recognise that requirements for measures to protect employee health and wellbeing can change | **Persuasive communication** | Guiding agents towards adoption of an idea, attitude, or action by using argument or other means | Messages should be relevant, not too different from individual’s beliefs. Can be stimulated by surprise and repetition | E.g., link to information on stroke survivor’s limitations changing over time. |
|  |  | Decide with relevant stakeholders how/when to record and review protective measures for employees’ health and safety | **Persuasive communication** | Guiding agents towards adoption of an idea, attitude, or action by using argument or other means | Messages should be relevant, not too different from individual’s beliefs. Can be stimulated by surprise and repetition | E.g., Guidance in legislation, HSE website, link to overview of stakeholder roles |
|  |  |  | **Environmental re-evaluation** | Encouraging realising the negative impact of the unhealthy behaviour (i.e., non-disclosure), and the positive impact of the healthy behaviour (i.e., disclosure) | Stimulation of both cognitive and affective appraisal to improve appraisal and empathy skills | E.g., Stories showing impact on employee health and wellbeing when regular reviews were done/not done. |
|  |  |  | **Active learning** | Encouraging learning from goal-driven and activity-based experience | Time, information and skills | E.g., Task – discussion with stakeholders and production of a template (or decision to amend or use one provided in toolkit) – e.g., risk assessment form, with timelines of when they should be completed. |
|  |  | Regularly record and review protective measures for employees’ health and safety  Analyse whether changes to protective measures for employees’ health and safety are needed | **Active learning** | Encouraging learning from goal-driven and activity-based experience | Time, information and skills | Reviews to be completed at timepoints specified above. Analysis of changes needed to be included on template form. |
| All steps | Relevant to all POs. Identify appropriate stakeholders and sources for support with (with consent from stroke survivor if needed). | Recognise that relevant stakeholders and organisations can advise on measures to protect health and safety of employees, and manage impact of the stroke survivor’s return (e.g., strategies to maintain productivity and maintain wellbeing) | **Persuasive communication** | Guiding agents towards adoption of an idea, attitude, or action by using argument or other means | Messages should be relevant, not too different from individual’s beliefs. Can be stimulated by surprise and repetition | Message: there are stakeholders and organisations than can help…provide link to overview of roles and organisation contact details  Benefits of liaising with stakeholders about health and safety measures, stories/quotes relating to RTW in different work industries and environments |
|  |  |  | **Arguments** | Using a set of one or more meaningful premises and a conclusion | For central processing of arguments they need to be new to the message receiver. |  |
|  | Relevant to all POs. Liaise with relevant stakeholders (including stroke survivor) or organisations, review information, or attend training. | Obtain contact details of stakeholders and organisations who can advise on measures to protect health and safety of employees |  |  |  |  |

| **Determinant: Intentions**  **(Stroke survivor may not know if they are ready to start preparing for return to work)** | | | | | | |
| --- | --- | --- | --- | --- | --- | --- |
| **Steps in intervention process** | **Performance objectives for stroke survivor** | **Change objectives** | **Theory-based methods** | **Definition** | **Parameters** | **Application (i.e., linked toolkit content)** |
| 1 | PO.1. Reflect on- and communicate readiness (to/with employer) to start planning for returning to work | Reflect on the benefits of returning to work (e.g., maintain financial income, maintain social relationships, sense of purpose and routine, etc) | **Persuasive communication** | Guiding agents towards adoption of an idea, attitude, or action by using argument or other means | Messages should be relevant, not too different from individual’s beliefs. Can be stimulated by surprise and repetition | Message: It is good to reflect on whether ready to start planning/preparing for RTW |
|  |  |  | **Active learning** | Encouraging learning from goal-driven and activity-based experience | Time, information, and skills | Exercise: Stroke survivor to complete pros and cons list for RTW. List could include pre-filled in examples, including potential impact of non-RTW on co-workers and family.  Goal: Reason/s why they want to RTW (to communicate with employer to show readiness to start preparing?) |
|  |  |  | **Environmental re-evaluation** | Encouraging realisation of negative impact of unhealthy behaviour (e.g., non-RTW), and positive impact of healthy behaviour (e.g., RTW) | Stimulation of cognitive and affective appraisal to improve appraisal and empathy skills |  |
|  |  | Reflect on aspects of the environment that could facilitate return to work (e.g., physical aspects like work environment, social support available [e.g., supportive co-workers], etc) | **Persuasive communication** | Guiding agents towards adoption of an idea, attitude, or action by using argument or other means | Messages should be relevant, not too different from individual’s beliefs. Can be stimulated by surprise and repetition | Message: It is good to reflect on whether ready to start planning/preparing for RTW |
|  |  |  | **Active learning** | Encouraging learning from goal-driven and activity-based experience | Time, information, and skills | Exercise: Stroke survivor to complete facilitators list for RTW. List could include pre-filled in examples, e.g., social/instrumental support from co-worker/s, option to work from home, take breaks when needed, etc.  Goal: List of potential facilitators that could be communicated to employer when stating readiness to start preparing for- and planning RTW |

| **Determinant: Knowledge**  **Stroke survivor:**  Stroke survivor may believe work caused their stroke, or worry about future impact of work on their health  Stroke survivor may not know if they are ready to start preparing for return to work  Stroke survivors don’t always know who/how to contact people that can help them understand their capabilities, limitations, and needs  Stroke survivors not aware of their capabilities and limitations  **Employer:**  Employers don’t always know who can help them with understanding stroke survivor’s abilities  Employers lack general knowledge of stroke causes and impact  Employers lack knowledge of specific impact of stroke on stroke survivor  Employers over-estimate stroke survivor abilities based on good pre-stroke abilities  Employers have limited knowledge of responsibilities relating to the return-to-work process, e.g., according to legal obligations, or organisational sick leave and procedures  Employers have pre-conceived beliefs about stroke and possibility of RTW | | | | | | |
| --- | --- | --- | --- | --- | --- | --- |
| **Steps in intervention process** | **Performance objectives for stroke survivor** | **Change objectives** | **Methods** | **Definition** | **Parameters** | **Application (i.e., linked toolkit content)** |
| 1 | PO.1. Reflect on- and communicate readiness (to/with employer) to start planning for returning to work | Obtain contact details and liaise with stakeholders (e.g., consultant, OT) who could educate them on the cause/s of their stroke, and advise on work participation | **Advance organisers** | Presenting an overview of the material than enables a learner to activate relevant schemas so that new material can be associated | Schematic representations of the content or guides to what is to be learned | E.g., Overview of stakeholders’ roles/skills accessible through menu of toolkit. Overview could include how their contact details may be obtained. Could also include list of helpful organisations. Refer to this overview throughout toolkit, highlight throughout toolkit in bold the stakeholders being mentioned that may be able to help at certain timepoints (and also highlight how they can help). |
|  |  |  | **Active learning** | Encouraging learning from goal-driven and activity-based experience | Time, information, and skills | Task: Look at overview of stakeholders and make list of who to contact and when re. learning causes of stroke |
|  |  | Recognise the following:  - A gradual approach to planning and returning to work is needed  - Their capabilities may not reach their pre-stroke level again (and that is okay)  - A trial and error approach will be needed  -They will need to participate in action planning at regular timepoints before, during, and after the return-to-work date | **Persuasive communication** | Guiding agents towards adoption of an idea, attitude, or action by using argument or other means | Messages should be relevant, not too different from individual’s beliefs. Can be stimulated by surprise and repetition | Message: see PO.  May also be included in role play videos. |
| 2 | PO.2. Appraise capabilities and limitations in relation to working role | Find out roles and contact details of relevant stakeholders (e.g., OT) who could help with appraisal  Contact stakeholders (including family and other  stroke survivors, employers or co-workers [i.e., ‘buddies’], if needed) for support with appraisal of limitations and needs, and communication of needs to employer. | See PO.1, row 1 (advance organisers and active learning – adapt to this action) | | | |
|  |  | Appraise abilities needed for working role  Appraise and compare current functional abilities to abilities needed for working role  Identify strengths and limitations for working role | **Persuasive communication** | Guiding agents towards adoption of an idea, attitude, or action by using argument or other means | Messages should be relevant, not too different from individual’s beliefs. Can be stimulated by surprise and repetition | Message: Including the right stakeholders in the appraisal process has many benefits (including planning communicating needs to employer). Refer to overview of stakeholder roles and role play videos showing this if relevant. |
|  |  |  | **Modelling** | Providing an appropriate model; being reinforced for the desired action | Attention, remembrance, self-efficacy and skills, reinforcement of model, identification with model, coping model instead of mastery model | E.g., Video interview with OT or stroke survivor talking about importance of appraising capabilities and needs for RTW early on, cite research evidence? |
|  | PO.3. Identify what is needed to enable work participation | Consider strengths and limitations, and identify needs (e.g., adjustments) for work participation  Decide which identified needs are essential for enabling or maintaining work participation |  |  |  |  |
|  |  |  | **Active learning** | Encouraging learning from goal-driven and activity-based experience | Time, information, and skills | Provide stroke survivor with tools as part of a 3-step appraisal process. Goal at end: a list of identified needs for work participation |
|  | PO.4. Consider what needs are essential for the employer to know for provision of support |  |  |  |  |  |
| 3 | PO.5. Clearly communicate essential needs to employer | Liaise with relevant stakeholders to plan how best to communicate essential needs to employer (e.g., face-to-face, in writing with copies for both) | **Active learning** | Encouraging learning from goal-driven and activity-based experience | Time, information, and skills | Task: Identify how they would like to communicate needs to employer. E.g., Write letter to employer with list of identified needs. Option to read letter or give it to employer to read. Provide template for letter and/or script to facilitate verbal disclosure of stroke and relay of needs |
| 5 | PO.6. Repeat PO. 2-5, regularly review needs with employer on ongoing basis, as agreed (e.g., monthly basis) | Recognise that limitations can change | **Persuasive communication** | Guiding agents towards adoption of an idea, attitude, or action by using argument or other means | Messages should be relevant, not too different from individual’s beliefs. Can be stimulated by surprise and repetition | E.g., Give examples from research literature, role play videos, re-emphasises initial recommendation that pre-stroke capabilities may never return |
| **Steps in intervention process** | **Performance objectives for employer** | **Change objectives** | **Methods** | **Definition** | **Parameters** | **Application (i.e., linked toolkit content)** |
|  |  |  |  |  |  |  |
| 1 | PO.1. Contact stroke survivor and jointly agree communication schedule | Recognise the potential benefits to liaising with a stroke survivor (and family) about their abilities  (e.g., for understanding their abilities and providing more tailored support) | **Persuasive communication** | Guiding agents towards adoption of an idea, attitude, or action by using argument or other means | Messages should be relevant, not too different from individual’s beliefs. Can be stimulated by surprise and repetition | E.g., Cite research evidence, role play videos showing benefits of liaison in real life experiences (including where stroke survivors successfully returned to work) |
|  |  |  | **Modelling** | Providing an appropriate model; being reinforced for the desired action | Attention, remembrance, self-efficacy and skills, reinforcement of model, identification with model, coping model instead of mastery model | Video from employer discussing benefits of liaising with stroke survivor about their abilities |
|  |  | Recognise the importance and potential benefits of early, regular communication with employees post-stroke, as recommended by the Stroke Association**, Acas***, CIPD****, and SOM | **Persuasive communication** | Guiding agents towards adoption of an idea, attitude, or action by using argument or other means | Messages should be relevant, not too different from individual’s beliefs. Can be stimulated by surprise and repetition | Message: See PO.  Cite guidance/research evidence. E.g., link to greater likelihood of retaining stroke survivor in organisation |
|  | PO.2. Recognise limited general knowledge of stroke. | Recognise that stroke affects individuals differently | **Persuasive communication** | Guiding agents towards adoption of an idea, attitude, or action by using argument or other means | Messages should be relevant, not too different from individual’s beliefs. Can be stimulated by surprise and repetition | Message: Stroke affects individuals differently, associated with more disabilities than any other condition  E.g., brief overview of different ways in which stroke can affect individual. Signpost to Stroke Association/Different Strokes information for further detail. |
|  |  | Evaluate general knowledge about stroke causes and impact | **Active learning** | Encouraging learning from goal-driven and activity-based experience | Time, information, and skills | 5-min quizzes, drag-and-drop activities, or reflective questions for employer to complete. Goal: Answers could show them if they have knowledge deficits, and show them what they need to learn more about. Toolkit provides tailored list of things to research as a result (e.g., signpost to Stroke charity websites) |
| 2 | PO.3. Recognise limited understanding of responsibilities during return-to-work process (according to legislation and organisational policies and procedures) | Evaluate knowledge of responsibilities relating to the return-to-work process (e.g., making reasonable adjustments, informing them about resources available) |  |  |  |  |
|  | PO.4. Recognise limited understanding of organisational resources available to stroke survivor employee | Evaluate knowledge of internal and external resources available to support stroke survivor employee | **Advance organisers** | Presenting an overview of the material than enables a learner to activate relevant schemas so that new material can be associated | Schematic representations of the content or guides to what is to be learned | e.g., link to overview page of relevant stakeholders and organisations, where info on their role/resources could be included, along with contact details (where possible). Highlight in bold the helpful things stakeholders could do (as in previous sections) |
|  |  |  | **Active learning** | Encouraging learning from goal-driven and activity-based experience | Time, information, and skills | Task: Compile list of resources with goal to present it to stroke survivor (template could be provided for this). Could be a drag-and-drop exercise with list compiled by toolkit at end (that they can download/edit). |
| 3 | PO.5. Recognise limited knowledge of specific impact of stroke on stroke survivor. | Evaluate knowledge about specific impact of stroke on stroke survivor | **Active learning** | Encouraging learning from goal-driven and activity-based experience | Time, information, and skills | Drag-and-drop activity, or editable template to record disabilities (e.g., like a bodymap) – could be done with stroke survivor during reviews  Goal: Identify things they need to learn more about (e.g., if they need to learn about impact more, or whether they need to learn more about specific issues, e.g., visual problems) |
| 4 | PO.7. Recognise need for support with identifying and organising reasonable adjustments for stroke survivors, that are affordable and don’t put any employees’ health and safety at risk. | Recognise that provision of reasonable adjustments is their responsibility according to the Equality Act 2010 | **Persuasive communication** | Guiding agents towards adoption of an idea, attitude, or action by using argument or other means | Messages should be relevant, not too different from individual’s beliefs. Can be stimulated by surprise and repetition | e.g., define what disability and reasonable adjustments are, according to Equality Act. Clear and concise. Signpost elsewhere for further info, e.g., Acas. |
|  |  |  | **Active learning** | Encouraging learning from goal-driven and activity-based experience | Time, information, and skills | E.g., Brief activity. Give two scenarios, ask employer to pick option on what is correct and what isn’t according to legislation, e.g., definition of whether adjustment is “reasonable or not,” or opinion on whether employer did right behaviour (give good and bad examples) |
|  |  |  |  |  |  | E.g., input stroke survivor disabilities into tool, receive list of potential reasonable adjustments to consider (with liaison with relevant stakeholders) |
| 5 | PO.8. Regularly review stroke survivor’s needs (with stroke survivor) on ongoing basis as agreed (e.g., monthly basis), repeat PO. 5-7 | Recognise that a stroke survivor’s limitations can change | **Persuasive communication** | Guiding agents towards adoption of an idea, attitude, or action by using argument or other means | Messages should be relevant, not too different from individual’s beliefs. Can be stimulated by surprise and repetition | E.g., Give examples from research literature, role play videos, re-emphasises initial recommendation that pre-stroke capabilities may never return |
| All steps | Relevant to all POs. Identify appropriate stakeholders and sources for support with (with consent from stroke survivor if needed). | Recognise that there are stakeholders, educational sources and/or training that can improve general knowledge of stroke  Recognise that there are stakeholders, educational sources and/or training that can improve knowledge of specific impacts of stroke (e.g., fatigue)  Recognise that there are stakeholders, informational sources, and/or training that can improve knowledge of employer responsibilities relating to the return-to-work process | **Advance organisers** | Presenting an overview of the material than enables a learner to activate relevant schemas so that new material can be associated | Schematic representations of the content or guides to what is to be learned | e.g., link to overview page of relevant stakeholders and organisations, where info on their role/resources could be included, along with contact details (where possible).  Give message about other stakeholders and organisations being able to help with these things. Highlight in bold in section the helpful things stakeholders could do (as in previous sections) |
|  | Relevant to all POs. Liaise with relevant stakeholders (including stroke survivor) or organisations, review information, or attend training. | Obtain contact details of stakeholders who can help with understanding stroke survivor’s abilities (with consent from stroke survivor)  Obtain contact details of stakeholders, collate educational information, and/or organise training to improve general knowledge of stroke  Obtain contact details of stakeholders, collate educational information and/or organise training to improve knowledge of specific impacts of stroke (e.g., fatigue)  Obtain contact details of stakeholders, collate information and/or organise training to improve knowledge of employer responsibilities relating to the return-to-work process  Obtain contact details of stakeholders, collate educational information, and/or organise training to improve skills and confidence for providing reasonable adjustments and carrying out RTW process actions | **Persuasive communication** | Guiding agents towards adoption of an idea, attitude, or action by using argument or other means | Messages should be relevant, not too different from individual’s beliefs. Can be stimulated by surprise and repetition | E.g., Cite research evidence, role play videos showing benefits of liaison in real life experiences |
|  |  |  | **Active learning** | Encouraging learning from goal-driven and activity-based experience | Time, information, and skills | 10-min task at end of each step: Compile to-do list of people to contact about certain things (e.g., support, information/advice, training).  Goal: contact them as and when needed (with stroke survivor consent if needed) |
|  |  |  | **Persuasive communication** | Guiding agents towards adoption of an idea, attitude, or action by using argument or other means | Messages should be relevant, not too different from individual’s beliefs. Can be stimulated by surprise and repetition | E.g., Cite research evidence, role play videos showing benefits of liaison in real life experiences |
|  |  |  | **Modelling** | Providing an appropriate model; being reinforced for the desired action | Attention, remembrance, self-efficacy and skills, reinforcement of model, identification with model, coping model instead of mastery model | Video from employer discussing benefits of liaising with different stakeholders about different things (see POs) |

| **Determinant: Social/Professional Role & Identity**  **(Employers not always willing to engage in communications about stroke survivors’ abilities,**  **e.g., due to perceptions that it is role of HR or OH)** | | | | | | | | | |
| --- | --- | --- | --- | --- | --- | --- | --- | --- | --- |
| **Steps in intervention process** | **Performance objectives for employer** | **Change objectives** | **Methods** | **Definition** | | | **Parameters** | | **Application (i.e., linked toolkit content)** |
| 4 | PO.7. Recognise need for support with identifying and organising reasonable adjustments for stroke survivors, that are affordable and don’t put any employees’ health and safety at risk. | Recognise the potential benefits to liaising with a stroke survivor (and family) about their abilities (e.g., for understanding their abilities and providing more tailored support) | **Persuasive communication** | Guiding agents towards adoption of an idea, attitude, or action by using argument or other means | | | Messages should be relevant, not too different from individual’s beliefs. Can be stimulated by surprise and repetition. Will include arguments. | | Information on why important to understand stroke survivor abilities (cite research literature/guidance/legislation)  May also be included in videos of stroke survivors'/employers’ RTW experiences |
|  |  |  | **Modelling** | Providing an appropriate model; being reinforced for their desired action | | | Attention, remembrance, self-efficacy, and skills; reinforcement of model; coping model instead of mastery model | | See above – e.g., research evidence/videos/written narratives showing what happened when employers liaised (or did not liaise) with stroke survivors about their abilities |
|  |  |  | **Information about others’ approval** | Providing information about what others think about the person’s behaviour and whether others will approve or disapprove of any proposed behaviour change | | | Positive expectations are available in the environment  E.g., work well in organisations where employee health/wellbeing and retention are valued | | E.g., Cite relevant qualitative research literature, include in videos of stroke survivor/employers’ RTW experiences. Cite guidance from CIPD, Stroke Association, etc. |
|  |  |  | **Mobilising social support** | Prompting communication about behaviour change in order to provide instrumental and emotional social support | | | Combines caring, trust, openness, and acceptance with support for behavioural change; positive support is available in the environment | | E.g., Coalition/network of RTW ambassadors, discussion session on importance- and strategies for liaising with stroke survivors about RTW and abilities |
|  |  | Recognise that other stakeholders (e.g., occupational therapists) are specially trained to recommend reasonable adjustments in line with stroke survivors’ abilities | **Persuasive communication** | Guiding agents towards adoption of an idea, attitude, or action by using argument or other means | | | Messages should be relevant, not too different from individual’s beliefs. Can be stimulated by surprise and repetition | | See above – e.g., research evidence/videos/written narratives showing what happened when employers liaised (or did not liaise) with relevant stakeholders about reasonable adjustments |
|  |  |  | **Individualisation** | Providing opportunities for learners to have personal questions answered or instructions paced according to their individual progress | | | Personal communication that responds to a learner’s needs | | E.g., Tool for matching stroke survivor limitations with potential reasonable adjustments. Information provided could suggest stakeholders who may be specially trained to support with that type of reasonable adjustment (e.g., health and safety officer, occupational therapist). Could refer to signposting section to organisations who could advise on gaining stakeholder input (e.g., RCOT, HSE) |
|  |  |  | **Mobilising social support** | Prompting communication about behaviour change in order to provide instrumental and emotional social support | | | Combines caring, trust, openness, and acceptance with support for behavioural change; positive support is available in the environment | | E.g., Coalition/network of RTW ambassadors, discussion session including experiences liaising with relevant stakeholders who could help with identifying and organising reasonable adjustments (e.g., what they did, how they helped, signposting to individual professionals or organisations) |
| All steps | Relevant to all POs. Identify appropriate stakeholders and sources for support (with consent from stroke survivor if needed). | Recognise importance of knowing specific impact of stroke on stroke survivor (i.e., for own role in providing support) | See all of previous table content above | | | | | | |
|  |  |  | **Persuasive communication** | | Guiding agents towards adoption of an idea, attitude, or action by using argument or other means | | Messages should be relevant, not too different from individual’s beliefs. Can be stimulated by surprise and repetition | | E.g., Messages throughout all steps – emphasise importance of knowing specific impact of stroke on stroke survivor |
|  |  | Recognise that external stakeholders may know more about a stroke survivor’s abilities than the organisation | See “Recognise that other stakeholders (e.g., occupational therapists) are specially trained to recommend reasonable adjustments in line with stroke survivors’ abilities” table content above. In content mention when stakeholders were external to the organisation. | | | | | | |
|  |  |  | **Persuasive communication** | Guiding agents towards adoption of an idea, attitude, or action by using argument or other means | | | Messages should be relevant, not too different from individual’s beliefs. Can be stimulated by surprise and repetition | | E.g., in each intervention step, give box with brief message stating which external stakeholders (e.g., occupational therapist) may be involved at that point, who may know and be able to advise on the stroke survivor's abilities, refer to signposting section on contact details for relevant organisations |
|  | Relevant to all POs. Liaise with relevant stakeholders (including stroke survivor) or organisations, review information, or attend training. | Recognise the potential benefits to liaising with a stroke survivor (and family) about their abilities  (e.g., for understanding their abilities and providing more tailored support) | See “Recognise the potential benefits to liaising with a stroke survivor (and family) about their abilities (e.g., for understanding their abilities and providing more tailored support)” for step 4 (PO.7.) above. | | | | | | |
|  |  |  | **Persuasive communication** | Guiding agents towards adoption of an idea, attitude, or action by using argument or other means | | | Messages should be relevant, not too different from individual’s beliefs. Can be stimulated by surprise and repetition | | E.g., Messages throughout all steps – briefly emphasise importance of liaising with stroke survivor (and family – with consent from stroke survivor) (refer to step 4 for further info on why liaison with stroke survivor is beneficial) |
|  |  | Recognise importance of knowing specific impact of stroke on stroke survivor (i.e., for own role in providing support) | **Persuasive communication** | Guiding agents towards adoption of an idea, attitude, or action by using argument or other means | | | Messages should be relevant, not too different from individual’s beliefs. Can be stimulated by surprise and repetition | | E.g., Messages throughout all steps – emphasise importance of knowing specific impact of stroke on stroke survivor (refer to step 4 for further info on why liaison with stroke survivor is beneficial) |
|  |  | Recognise that other stakeholders (e.g., occupational therapists) are specially trained to recommend reasonable adjustments in line with stroke survivors’ abilities | See “Recognise that other stakeholders (e.g., occupational therapists) are specially trained to recommend reasonable adjustments in line with stroke survivors’ abilities” table content above. In content mention when stakeholders were external to the organisation. | | | | | | |
|  |  |  | **Persuasive communication** | Guiding agents towards adoption of an idea, attitude, or action by using argument or other means | | Messages should be relevant, not too different from individual’s beliefs. Can be stimulated by surprise and repetition | | E.g., Messages throughout all steps – e.g., examples of stakeholders who might be able to support at that point. Could refer to adjustment matching tool in step 4, also refer to signposting section where there could be details of organisations with educational/advisory resources and training providers (including contact details)  briefly emphasise importance of liaising with stroke survivor (and family – with consent from stroke survivor) (refer to step 4 for further info on why liaison with stroke survivor is beneficial)  Could include signposting to organisations who could advise on gaining stakeholder input (e.g., RCOT, HSE) | |
|  |  | Recognise that external stakeholders may know more about a stroke survivor’s abilities than the organisation | Same as row above. Ensure all messages mention where stakeholders may be external to organisation. | | | | | | |

1. This table shows the research evidence regarding similar interventions. It details the effective and/or acceptable practical applications used in these interventions (that relate to the determinants and performance objectives identified in our needs assessment).

| **First author**  **Year of publication** | **Focus of intervention,**  **effectiveness findings** | **Employee with injury or health condition** | | **Employer** | |
| --- | --- | --- | --- | --- | --- |
|  |  | **Relevant determinants** | **Findings relating to applications/methods used** | **Relevant determinants** | **Findings relating to applications/methods used** |
| Svanholm 2023 [3] | eHealth intervention, Sustainable Worker Digital Support for Persons with Chronic Pain and Their Employers (SWEPPE)  Not yet tested in clinical trial | Beliefs about capabilities | Before intervention: Unsure they had the ability to identify goals and balance goal-focused work with recovery | Knowledge | Text aimed at the right level (with reasonable length) perceived as being effective for learning about pain, its consequences, and the need for adaptations in work and everyday life.  Employers wanted more concrete examples in the library of information.  Good to help employers understand complexity of health condition and that rehabilitation is a long process. |
|  |  | Knowledge | Films useful for those who found it difficult to read and assimilate text. |  |  |
|  |  | Behavioural regulation/ Knowledge | Self-rating and self-monitoring tools helped them understand their health and behaviours (e.g., understanding relationship between pain and stress). This made it easier for them to understand their needs, plan strategies and activities and be kind to themselves. This also facilitated acceptance for one ppt. Doing this daily became tedious though.  Would be useful to be able to plan activities via the self-monitoring and self-rating graphs, with a calendar (aiming for prevention of pain). Would have preferred someone to talk to about the graphs re. interpretation.  Appreciated ability to write own strategies, choose variables to be presented on graphs, and make personal notes. Wanted to be able to select variables to be shown on graphs.  Action plan useful for defining strategies and goals |  |  |
|  |  |  |  | Skills/Beliefs about Capabilities | Concerns about goals not being specific enough, or not understanding information in library. Support from a health professional considered necessary for this. |
|  |  | General opinion on design and implementation | Linked medical programme (interdisciplinary pain rehab program run by health professionals) vital for trustworthiness of intervention  Flexibility, precision, and tailoring were valued. Also good to have paper form of intervention in case phone use too energy consuming. | General opinion on design and implementation | Linked medical programme (interdisciplinary pain rehab program run by health professionals) vital for trustworthiness of intervention. Would be useful to have reminders to use the intervention. |
|  |  | Beliefs about capabilities  Beliefs about consequences/ Emotions | Useful that intervention provided support with analysing a situation and a strategy for “doing better.” |  |  |
| Greidanus 2021 [4] | Website intervention targeted at employers. Objective: to enhance successful RTW of cancer survivors.  Not effective in feasibility study. Issues with recruitment in this phase means definitive RCT not possible. |  |  | General opinion on design and implementation | Liked clear structure and layout. Intervention (website) was easily accessible. Combination of visual and textual content appealing.  Didn’t like when scenarios unrealistically positive or assumptions made (e.g., all conversations take place F2F in office). Non-users missed the email with the website link, so didn’t know of its existence. Others made aware through HR/OH staff. Not all intervention stages relevant (e.g., not all employees wanted to consider RTW when undergoing treatment). Sometimes IT system blocked website, videos sometimes lagged, and parts of intervention not always visible on smaller screens. |
|  |  |  |  | Skills/Beliefs about capabilities | Useful parts of the intervention:  -textual tips per RTW phase (clear about keeping in touch)  -conversation checklists (provide structure, overview, guidance)  -communication videos |
|  |  |  |  | Social/Professional Role & Identity/  Knowledge | -visual content showed importance of listening to employee with cancer and realising there are different types of cancer survivors (e.g., non-cooperative, emotional, etc). |
|  |  |  |  | Knowledge | Suggestions for improvement:  -clear intro/overview at start  -general info about health condition and relevant legislation |
|  |  |  |  | Social/Professional Role & Identity | -suggestions for allocation of tasks across staff (e.g., HR, manager, OH) |
|  |  |  |  | Implementation | -Forum to consult with fellow employers  -Access to specialised coach for complex scenarios |
|  |  |  |  | Knowledge/ Skills/ Beliefs about Capabilities | Increased knowledge and awareness of employees’ experiences and how to act/what to say naturally increased employer skills (or confirmed what they already knew). |
| Volker 2017 [5] | Blended web-based intervention with 2 parts:  1.eHealth module for employee with CMD. Aim: to change their cognitions re. RTW  2.Email decision aid for OH with advice re. treatment and referral options (based on employee treatment progress)  Statistically significantly shorter median duration until first RTW in intervention group. At 9 months post-baseline: significantly more ppts in intervention group achieved remission. | Beliefs about capabilities/ Beliefs about Consequences/ Emotions/ Intentions/ Knowledge | Liked the following parts:  -info about positive/negative thoughts with regard to RTW while having symptoms  -learning problem-solving skills  -receiving advice about physical complaints | Implementation | Issues: Did not know employee taking part in intervention, or missed/forgot about emails from decision aid.  Email messages from decision aid supporting them in guiding employee. Emails had sufficient info and were visually attractive.  Comforting having opportunity to contact a psychiatrist if needed.  Potential issues with implementation:  -limited time of OH (consultations are low frequency and short duration)  -email contact not typically provided between employee and OH between consultations |
|  |  | General opinion on design | Disliked the following:  -too little guidance/contact, feedback and personal attention  -content too general or N/A to their scenario or symptoms  Suggestions for improvement:  -more contact with researchers or OH  -reminders to continue intervention  -not repeatedly asking same questions |  |  |
| Schumacher 2017 [6] | Self-guided WorkPlan workbook designed to support cancer survivors with RTW (e.g., with fortnightly telephone calls to discuss progress).  Feasibility study confirmed the feasibility of a definitive RCT. E.g., at 6 months, 30% of the control group had RTW, compared with 43% of intervention group. | Behavioural regulation/ Beliefs about Capabilities/ Beliefs about Consequences/ Emotions/ Intentions/ Knowledge | Employees felt overwhelmed at start of RTW process. Found it empowering to set goals, identify barriers, and to reflect on the small details that would make RTW manageable. |  |  |
|  |  | Beliefs about Consequences/ Emotions | The act of writing helped them focus their thoughts about work and enabled them to brainstorm ideas to solve RTW challenges (e.g., by writing down different options, evaluating how effective they would be). They liked noting down their thoughts and emotions and taking ownership of them, helped alleviate anxiety about RTW. |  |  |
|  |  | Beliefs about Consequences/ Emotions/ Knowledge | Final task of constructing RTW plan (e.g., specifying desired date of RTW, hours/workdays, concerns or support needs, specification of work tasks able (and not able) to do. This level of specificity helped alleviate anxiety about RTW. |  |  |
|  |  | Behavioural regulation/ Beliefs about Consequences/ Emotion/ Knowledge | Found it useful that the intervention enabled them to imagine problems that may arise, and making plans to lessen or prevent their impact on work. E.g., considering all daily work tasks, potential events at work (e.g., coping with co-workers’ reactions), and how they might respond. This also enabled them to do mental role play, rehearsing responses and gaining confidence in their abilities. |  |  |
|  |  | Knowledge | Helpful to have list of things they could say to employer. Some had not realised it could be beneficial communicating with employer, that they could initiate the conversations, or that the employers and co-workers would want to listen- and speak with them. The RTW plan worksheet was an excellent tool for guiding communication with the employer (and for joint planning sessions with the employer) (something they could print off and take with them). Knowing how to (and what to) convey re. their specific RTE needs increased employees’ confidence. Types of things they discussed: Risk assessments before RTW, details of phased RTW, how the RTW process would be managed, tasks they would need support with, how their support plans would be monitored and assessed. |  |  |
| Cadilhac 2020 [7] | Personalised electronic self-management intervention to support person-centre goal attainment and secondary prevention post-stroke.  Goal attainment in intervention group achieved for goals relating to function, environment, and participation (control: environment only). | Behavioural regulation/ Knowledge | Goal setting form helpful in developing goals. Also helpful when they consulted clinicians about goals. |  |  |
|  |  | Implementation | Electronic messages reportedly assisted intervention participants in achieving goals. |  |  |

**References**

1. Bartholomew-Eldridge L, Markham C, Ruiter R, Fernández M, Kok G, Parcel. Planning health promotion programs: An intervention mapping approach. 4th ed. San Francisco, CA: Jossey-Bass; 2016.

2. Cane J, O'Connor D, Michie S. Validation of the theoretical domains framework for use in behaviour change and implementation research. Implementation science 2012;7(1):37-.

3. Svanholm F, Turesson C, Löfgren M, Björk M. Acceptability of the eHealth Intervention Sustainable Worker Digital Support for Persons With Chronic Pain and Their Employers (SWEPPE): Questionnaire and Interview Study. JMIR human factors. 2023;10:e46878-e.

4. Greidanus MA, de Rijk AE, Frings-Dresen MHW, Tiedtke CM, Brouwers S, de Boer AGEM, et al. The Use and Perceived Usefulness of an Online Toolbox Targeted at Employers (MiLES Intervention) for Enhancing Successful Return to Work of Cancer Survivors. Journal of occupational rehabilitation. 2020.

5. Volker D, Zijlstra-Vlasveld MC, Brouwers EPM, van der Feltz-Cornelis CM. Process evaluation of a blended web-based intervention on return to work for sick-listed employees with common mental health problems in the occupational health setting. Journal of occupational rehabilitation. 2017;27(2):186-94.

6. Schumacher L, Armaou M, Rolf P, Sadhra S, Sutton AJ, Zarkar A, et al. Usefulness and engagement with a guided workbook intervention (WorkPlan) to support work related goals among cancer survivors. BMC psychology. 2017;5(1):34.

7. Cadilhac DA, Andrew NE, Busingye D, Cameron J, Thrift AG, Purvis T, et al. Pilot randomised clinical trial of an eHealth, self-management support intervention (iVERVE) for stroke: feasibility assessment in survivors 12–24 months post-event. Pilot and feasibility studies. 2020;6(1):1-172.
