# Supplementary material for: Development of a digital, self-guided return-to-work toolkit for stroke survivors and employers using intervention mapping
Source: PLOS Digit Health. 2025 Aug 6;4(8):e0000971. doi: 10.1371/journal.pdig.0000971 (PMC12327610; doi:10.1371/journal.pdig.0000971)
Supplement: S6 Table — (DOCX) [file pdig.0000971.s009.docx]

**S6. Selection of practical applications.**

This document contains tables incorporating findings from previous research studies re. eHealth RTW interventions, theory-based methods for TDF determinants (mapped using the IM taxonomies), and ideas from the expert advisory group and workshop participants. All information was combined to come up with/add to/support previous ideas for appropriate applications that are theory- and evidence-based, and attractive to future participants.

General ideas based on research evidence:

- Clear overview at beginning of toolkit
- Video/text summaries and textual tips at end of each section [1, 2]
- Good to have downloadable PDF version (to print) in case too tiring to use electronic version [1]
- Make toolkit as applicable as possible to symptoms, i.e., tailored, contact with OH or researchers where possible [3]
- Do not repeatedly ask the same questions [3]
- Reminders to continue intervention [1, 3]
- Electronic messages help participants achieve goals [4]
- Toolkit needs clear structure and layout, combination of visual and textual information [2]
- Scenarios should not be unrealistically positive or assumptions made about how things are/could be done in an organisation [2]
- Toolkit should not be advertised (or link provided) by email only, as people have missed emails in other research [2, 3]
- Potential for IT issues. E.g., IT system blocked, videos lagging, parts of intervention not visible on smaller screens [2]
- Forum to consult with fellow employers (workshop participants also said this) [2]
- Access to specialised coach for complex scenarios [2]

| **STROKE SURVIVOR** | | | | | |
| --- | --- | --- | --- | --- | --- |
| **Step** | **Performance objectives** | **Determinants** | **Method** | **Practical strategy**  **(i.e., application)** | **Parameters for success** |
| Relevant to all steps | N/A | N/A | Advance organisers | Overview of stakeholders’ roles/skills. Include how contact details may be obtained, and list of helpful organisations. Refer to this overview throughout toolkit, highlight in bold the stakeholders being mentioned that may be able to help at certain timepoints (and also highlight how they can help). | Schematic representations of the content or guides to what is to be learned (e.g., use of bold font and/or colour to highlight) |
| 1 | PO.1. Reflect on- and communicate readiness (to/with employer) to start planning for returning to work | Beliefs about capabilities/Knowledge, Beliefs about consequences/ Emotion | Modelling  Persuasive communication  Verbal persuasion  Cultural similarity | Show how stroke survivors have successfully managed RTW (with no negative impact on health) by taking a gradual, trial-and-error approach to planning and returning, realising their capabilities may not reach pre-stroke level, and taking part in goal setting [1] and action planning at regular timepoints. E.g., Results from stroke VR/RTW studies, videos of stroke survivors telling of their experience relating to this, and written stories with image. Include emotional responses experienced by stroke survivors and strategies that helped *. | Stroke survivors need self-efficacy, and attention and memory capabilities.  Credible sources should be used for real life examples, conveying coping models rather than mastery models. Messages should be delivered by other stroke survivors, be relevant, and not too different from stroke survivor participants’ beliefs. To increase relevance, range of stroke survivors with different disabilities, ethnicities, ages, occupational roles, industries, etc, could be included in examples. |
|  |  | Beliefs about consequences/ Emotions/ Knowledge/ Intentions | Persuasive communication | Message 1:  It is good to reflect on whether read to start planning/preparing to RTW  Message 2: State which stakeholders/others may be able to educate and advise on cause/s of stroke and advise on RTW. Information on causes of most strokes (%). Include health psychologists as one of stakeholders mentioned (e.g. in relation to managing distressing thoughts).  Message 3: Preparing and planning for work involves gradual trial-and-error approach, and action planning at regular timepoints. | Messages should be relevant to stroke survivor (e.g., not focused only at those with particular types of stroke), or occupational role/organisation. |
|  |  |  | Active learning | Tasks:  1.Identify and make list of stakeholders to contact to understand more about cause/s of stroke and advice for work participation (if possible). Then to populate template based on their responses.  2.Reflective tool - writing down thoughts and emotions relating to RTW [5], to discuss with a professional.  3.Checklist to tick whether they feel willing and able to undertake certain aspects of RTW process (e.g., trial-and-error approach) | Stroke survivors need time, information (included in overview of stakeholders), and capabilities to complete these tasks. |
|  |  |  | Active learning | Guidance on communicating readiness to employer (e.g., conversation script)* Template to write own script [1, 5] Goal: to pass on to/communicate with employer | Stroke survivors need time, information (included in stakeholder overview), and capabilities to complete these tasks. |
|  |  | Knowledge, Intentions | Active learning  Environmental re-evaluation | 1.Pros and cons list for returning to work*  2.List of potential facilitators in environment.  Include pre-filled in examples. Aim: list of reasons why they want to RTW. | Stroke survivor needs time, information and skills (e.g., ability to do cognitive and affective appraisal) to construct list. |
| 2 | PO.2. Appraise capabilities and limitations in relation to working role | Knowledge | Modelling | Video interview with OT or stroke survivor talking about importance of appraising capabilities and needs for RTW early on. | Stroke survivor viewers needs to have self-efficacy, attention and memory capabilities. Coping models conveyed rather than mastery models. |
|  |  |  | Persuasive communication | Message: Including the right stakeholders in the appraisal process has many benefits (including planning communicating needs to employer). Refer to overview of stakeholder roles and role play videos showing this if relevant. | Messages should be relevant to stroke survivor, not too different from their beliefs. |
|  |  |  | Active learning | Goal-directed tasks:  1. Look at overview of stakeholders and make list of who to contact for support with appraisal of limitations and needs, and communication of needs to employer  2. Diary for stroke survivors (e.g., self-rating or self-monitoring tool) to document certain symptoms and identify triggers – this should be reviewed regularly* [1]  3. Comprehensive checklist of things to consider (e.g., getting to/from toilet) * | Stroke survivors need time, information (included in overview of stakeholders), and capabilities to complete these tasks. |
|  | PO.3. Identify what is needed to enable work participation. | Knowledge | Active learning  Tailoring | 3-step process and templates for identifying needs (. Include:  1 and 2. Job analysis tool (e.g., what job role requires capability-wise, identify barriers)  3. Come up with potential strategies/solutions) [1, 5]. Goal-directed task: enter in limitations into interactive tool – get list of tailored recommended adjustments*  Include note/signposting on potential for Access to Work to provide financial aid.* | Stroke survivors need time, information (e.g., job role description), and capabilities to complete these tasks. |
|  | PO.4. Consider what needs are essential for the employer to know for provision of support | Knowledge | Active learning | Reflective prompts to aid decisions on which needs are essential for enabling and maintaining work participation (e.g., refer to list of strategies and possible adjustments)*  Goal-directed task: highlight on list of recommended adjustments which ones are essential/desired to highlight to employer. | Stroke survivors need time, information (e.g., list of adjustments from PO.3 action), and capabilities to complete these tasks. |
| 3 | PO.5. Clearly communicate essential needs to employer | Beliefs about consequences/ Emotions, Knowledge | Arguments  Environmental re-evaluation | Message 1: Potential benefits for disclosure (e.g., give information on stroke survivor’s legal rights*, better return-on-investment to retain stroke survivor*) and what can happen if do not disclose. Include reference to overview of stakeholders, e.g., who may be able to support or advise on disclosure.  Message 2: Open communication about limitations may help maintain relationships with employer and co-workers. Include what may happen if communication not open. | Information in message needs to be novel to stroke survivor. Messages needs to stimulate the stroke survivor to make cognitive and affective appraisal. |
|  |  |  | Modelling | Videos or testimonials stroke survivors discussing positive impact of (early) disclosure to employer, and related benefits* [1], and open communication in maintaining relationships in workplace. | Stroke survivor viewers needs to have self-efficacy, attention and memory capabilities. Coping models conveyed rather than mastery models. |
|  |  |  | Active learning | Guidance on communicating with employer and co-workers (e.g., conversation script to use (“these are my current abilities, limitations, and needs”)*, template to write down own script [5] and list of identified needs (i.e., adjustments) | Stroke survivors need time, information (e.g., list of adjustments from PO.3 action), and capabilities to complete these tasks. |
| 4 | PO.6. Plan initial reasonable adjustments with employer and record them | Behavioural regulation/ Knowledge/ Beliefs about capabilities | Participation | Message: Some adjustments may not be realised until after RTW*, engagement of all relevant  stakeholders important in decision-making and problem-solving. Include Suggestions for simulation of work tasks to trial adjustments prior to RTW* | All participants need to be accepted as having high influence. Participants need to be motivated and have sufficient skills. |
|  |  |  | Active learning  Planning coping responses | Task: Include template to note down where problems might arise upon RTW (e.g., co-worker reactions, daily work tasks), note down strategies and rehearse how stroke survivor and employer might respond. [5]. | Stroke survivors need time, information (e.g., completed RTW plan), and capabilities to complete these tasks. Example written scenarios (e.g., co-workers’ negative reactions) needed to prompt reflection on potential issues that may arise. |
|  |  |  | Active learning  Public commitment | Suggestions and templates to aid planning tasks, e.g.:  -risk assessments before RTW  -workplace buddy to help with feedback and appraisal (if stroke survivor amenable to this)  -reasonable adjustments passport and linked goals (e.g., who will do what by when) [4, 5]  -wellbeing action plan  -RTW plan (specifying desired date of RTW, hours/workdays, concerns or support needs, specification of work tasks able (and not able) to do) [5]  -minutes to be made during meetings*  Mention overview of stakeholders, e.g., who may be able to support use of these tools if needed. | Stroke survivors need time, information (e.g., list of adjustments from PO.3 action), and capabilities to complete these tasks.  Public commitment: planned actions (i.e., goals) need to be announced publicly to those involved (with stroke survivor consent) |
| 5 | PO.7. Regularly review needs with employer on ongoing basis as agreed (e.g., monthly basis) | Behavioural regulation, Knowledge | Persuasive communication | Information that stroke survivor limitations can change over time. Give examples from research literature, videos of stroke survivors’ real life experiences, re-emphasise that pre-injury level of capabilities may never completely return. Examples of timelines, e.g., how often to complete reviews. | Messages should be relevant to stroke survivor, not too different from their beliefs. |
|  |  |  | Participation  Active learning  Public commitment  Implementation intentions | Task: Contract template to complete. Enter information on role of each participant (employer/stroke survivor) in review process (give examples). Ask each participant to sign this document at the end to signify that they have read and understood their role in regular reviews. Additional task at end: Book in series of regular review meetings into work calendars, set up calendar alerts. | Employer and stroke survivor need to be motivated to do regular reviews, and be willing to publicly commit to this via the signed contract. Both parties need time, information (e.g., access to work calendars) and adequate skill to do the tasks. |
|  |  |  | Active learning  Self-monitoring of behaviour  Goal setting | Task with employer (and any other stakeholders requested by stroke survivor, e.g., workplace buddy) (minutes to be made during meeting*): Look at reasonable adjustments passport (and feedback form if used) completed during step 4. Discuss previous adjustments and whether changes needed. Include reflective prompts to analyse any issues and come up with solution [1, 3]. Complete passport, including section at end with goals (e.g., who will do what by when) | Participants need time, information (e.g., reasonable adjustments passport), and capabilities to complete these tasks. Needs to be clear exactly what is being monitored (i.e., behaviours relating to specific adjustments) and reward should be reinforcing (e.g., positive feedback on stroke survivor’s effort and any progress made). Participants need to be committed to their goals, and goals must be difficult but attainable with their skill sets. |

*Indicates a suggestion by a workshop participant or expert advisory group member

| **EMPLOYER** | | | | | |
| --- | --- | --- | --- | --- | --- |
| **Step** | **Performance objectives** | **Determinants** | **Method**  **(citation/s for relevant theory)** | **Practical strategy**  **(i.e., application) (citation/s for research evidence)** | **Parameters for success** |
| 1 | PO.1. Contact stroke survivor and jointly agree communication schedule | Beliefs about consequences/ Emotions/ Knowledge | Persuasive communication  Arguments | Videos of successful real life RTW stories – stroke survivors talking about their experiences and how employers helped them (or didn’t) * Cite research evidence for further credibility.  Conclusive message: with the right support, stroke survivors can RTW. | Message needs to be novel to the employer, not too different from their beliefs (e.g., about possibility of stroke survivor successfully returning to work). |
|  |  |  | Persuasive communication  Modelling | Videos of employers discussing how they communicated with stroke survivors, and the benefits in doing so early on (and regularly) – e.g., in learning about their abilities. Show examples of good communication in videos (including importance of listening to stroke survivor) [2]. Cite relevant guidelines and research evidence. | Employers in videos need to convey coping strategies rather than mastery, range of employers included (e.g., self-employed, different industries or organisational sizes) (participants need to be able to identify with the employers in videos). Employer participants also need to have adequate attentional and memory capabilities for remembering messages given in videos.  Message should be relevant to employer, not too different from their beliefs (e.g., if they strongly believe that early and regular communication is inappropriate). |
|  |  | Skills/Beliefs about capabilities, Social/ Professional Role & Identity | Persuasive communication | Message: early communication with stroke survivors is important. Provide examples of things to consider and conversation starters*. |  |
|  |  |  | Active learning  Goal setting  Self-monitoring of behaviour  Planning coping responses | Series of brief tasks:  1.Reflective prompts re. confidence and skills for communicating with stroke survivor. If feel low in confidence or skills could consider tasks 3-5.  2. Complete first draft of communication schedule using template provided. Goal: take to first meeting with stroke survivor.  3.(Optional) make list of learning/training activities they will do. Refer to stakeholder overview for organisation details.  4. (Optional) Record learning/training activities in relevant organisational paperwork when completed (if available)  5. (Optional) List potential barriers to communicating with stroke survivors (e.g., not knowing what to do if they become upset). Plan and rehearse coping responses. | Employers need time, necessary information (e.g., conversation starters), and capabilities to complete tasks. Employers need to be committed to communicating with stroke survivor, and tasks achievable with their skill sets. If training, etc, recorded there must be a benefit to it being recorded (e.g., for performance review). For task 5, employers would need examples to prompt their listing of potential barriers. |
|  | PO.2. Recognise limited general knowledge of stroke. | Knowledge | Persuasive communication | Message: Stroke affects individuals differently, associated with more disabilities than any other condition. Further general information about stroke (e.g., cause/s, how it might look, e.g., disabilities, co-morbidities, and its potential impact on work abilities, and need for adaptations). Also information that rehabilitation can be a long process* | Message should be relevant to employer, not too different from their beliefs (e.g., if they strongly believe all stroke happen and affect individuals identically).  Text needs to be aimed at right level, reasonable length [1] |
|  |  |  | Active learning | 5-minute multiple-choice quiz or drag-and-drop exercise regarding general knowledge of stroke. Provide individualised list of things they may need to learn more about (based on their answers), with link to stakeholder overview containing organisational contact details (e.g., Stroke Association) for further information and/or training opportunities* | Employers need time, information (e.g., instructions), and capabilities to complete task. |
| 2 | PO.3. Recognise limited understanding of responsibilities during return-to-work process (according to legislation and organisational policies and procedures) | Knowledge, Social/Professional Role & Identity, Skills/ Beliefs about Capabilities | Persuasive communication  Chunking | Message 1. Overview of employers’ roles and responsibilities (e.g., legal obligation to provide reasonable adjustments) * (include suggestion to read organisational policies and procedures if available). Include acronym to aid memory on what makes adjustments reasonable.  Message 2. Information showing it is better return-on-investment for organisation to retain stroke survivor than replace them*.  Message 3. Flexibility is needed, include examples of strategies for dealing with environment and short staffing. * | Message should be relevant to employer, not too different from their beliefs (e.g., if they strongly believe stroke survivors cannot RTW post-stroke, or that it is others’ responsibility to support them).  Labels or acronyms assigned to material to aid memory.  Text needs to be aimed at right level, reasonable length [1] |
|  |  |  | Active learning  Goal setting  Self-monitoring of behaviour | 5-minute multiple-choice quiz or drag-and-drop exercise regarding employer role and responsibilities. Provide individualised list of things they may need to learn more about (based on their answers), with link to stakeholder overview containing organisational contact details (e.g., Stroke Association) for further information and/or training opportunities* (e.g., for information and advice about the employer’s role and responsibilities*).  Optional brief tasks:  1.Make list of learning/training activities they will do.  2. Record learning/training activities in relevant organisational paperwork when completed (if available) | Employers need time, information (e.g., instructions), and capabilities to complete task. Employers need to be committed to communicating fulfilling their role and responsibilities (e.g., it is their legal obligation), and tasks need to be achievable with their skill sets. If training, etc, recorded there must be a benefit to it being recorded (e.g., for performance review). |
|  | PO.4. Recognise limited understanding of organisational resources available to stroke survivor employee | Knowledge | Persuasive communication | Message: Internal and external resources may be available to improve support for the stroke survivor (give examples and link to overview of stakeholders and organisations) | Message should be relevant to employer, not too different from their beliefs (e.g., if they strongly believe resources are unavailable to them). |
|  |  |  | Active learning | Task: Compile list of resources with goal to present it to stroke survivor (template could be provided for this; include questions to help employer know what resources to consider or research in order to complete list). | Employers need time, information (e.g., instructions), and capabilities to complete task. |
| 3 | PO.5. Recognise limited knowledge of specific impact of stroke on stroke survivor. | Knowledge | Persuasive communication | Message: Re-emphasise that stroke affects individuals differently, with examples of residual limitations. E.g., 5-minute videos of stroke survivors talking about specific challenges faced during RTW (e.g., invisible disabilities) *. Include emotional responses experienced by stroke survivors and strategies that helped *. | Message should be relevant to employer, not too different from their beliefs (e.g., if they strongly believe stroke affects everyone identically, and that there are no emotional effects from stroke). |
|  |  |  | Active learning  Chunking | Task: Drag-and drop body map activity to ascertain whether they know if stroke survivor has certain limitations (e.g., “do they have difficulties remembering things?”). Responses of “I don’t know” lead to printable/downloadable list of individualised things they could find out in relation to stroke survivor. Include note on confidentiality and importance of consulting stroke survivor first. Use acronym to teach appropriate communication style. Include link to overview of stakeholders/organisations for further information about stroke-related residual limitations, training opportunities, and who to contact re. stroke survivor’s specific abilities and RTW prognosis (with stroke survivor consent). | Employers need time, information (e.g., body map template and associated questions), and capabilities to complete tasks.  Labels or acronyms assigned to material to aid memory. |
| 4 | PO.6. Recognise how stroke survivor’s return to work may impact wider team (e.g., who does tasks, how they’re done, co-workers’ feelings) | Beliefs about consequences/ Emotions/ Knowledge, Skills/Beliefs about capabilities | Persuasive communication  Modelling  Chunking | Guidance on how to assess whether RTW too risky (with examples of helpful strategies in different workplace environments). Include generic template for risk assessment, with guidance on how to make it bespoke. Also – include suggestion for suggestion for manager to review PEEP, fire evacuation, etc.* Signpost to HSE website in stakeholder/organisation overview *  Include guidance on how to approach discussion with stroke survivor and co-workers*. E.g., example phrases, tips on communication style (e.g., acronym to aid memory), video showing employer carrying out discussions with stroke survivor and co-workers in way that respects stroke survivor’s wishes regarding disclosure. | Message should be relevant to employer, not too different from their beliefs (e.g., if they strongly believe stroke affects everyone identically, and that there are no emotional effects from stroke).  Employers in videos need to convey coping strategies, range of employers included (e.g., self-employed, different industries or organisational sizes) (participants need to be able to identify with the employers in videos). Employer participants also need to have adequate attentional and memory capabilities.  Labels or acronyms assigned to material to aid memory. |
|  |  |  | Active learning  Planning coping responses | Task 1: Complete risk assessment (see above).  Task 2: Compile list of potential risks and discuss with stroke survivor and wider team (if appropriate). Goal: Ask for feedback and additional comments. Minutes to be made during meetings.  Task 3. Compile list of coping responses/strategies for high-risk situations, share and store appropriately. Refer to in future if needed. | Tasks 1 and 2 can be conjoined if appropriate and preferable (to save time). Employers need time, information (e.g., guidance and tools), and capabilities to complete tasks. |
|  | PO.7. Recognise need for support with identifying and organising reasonable adjustments for stroke survivors, that are affordable and don’t put any employees’ health and safety at risk. | Skills/Beliefs about capabilities, Knowledge | Persuasive communication | Message 1: Reminder message – i.e., define disability according to Equality Act, and what reasonable adjustments are (and that it is their responsibility to provide them). Link to stakeholder/organisation overview for further information. | Messages should be relevant to employer, not too different from their beliefs (e.g., if they strongly believe adjustments need to be fully identified prior to RTW). |
|  |  | Social/Professional Role & Identity, Knowledge | Modelling | Videos/written narratives showing benefits when employers liaised with stroke survivors and other stakeholders (e.g., occupational therapists) about their abilities. | Employers in videos need to convey coping strategies, range of employers included (e.g., self-employed, different industries or organisational sizes) (participants need to be able to identify with the employers in videos). Employer participants also need to have adequate attentional and memory capabilities. |
|  |  |  | Persuasive communication  Information about others’ approval | Message 2: It is important to understand stroke survivors’ unique (dis)abilities, so that suitable reasonable adjustments can be made and legal obligations met. Cite research evidence/ guidance in agreement with this. | Messages should be relevant to employer, not too different from their beliefs (e.g., if they strongly believe it is not their role to understand stroke survivors’ abilities). Positive expectations needed in organisational environment (e.g., where it might be expected that employers care about supporting employees with their health and wellbeing). |
|  |  | Skills/Beliefs about capabilities, Knowledge | Persuasive communication | Message 3: State that some adjustments may not be realised until after RTW *. Give examples of reasonable adjustments for different residual limitations (e.g., workplace buddy to help with feedback and appraisal) *. |  |
|  |  | Skills/Beliefs about capabilities, Knowledge, Social/Professional Role & Identity,  Beliefs about consequences/ Emotions | Persuasive communication | Message 4: The employer should offer every single active support they can under reasonable adjustments before reaching any conclusions about stroke survivors continuing on in the same work role – this conclusion should be made together (employer + employee) *. Refer to overview of stakeholders and organisations for support. |  |
|  |  | Skills/Beliefs about capabilities, Knowledge | Enactive mastery experience  Active learning | Task 1: Brief simulation/scenario exercises. Receive information about 3 different stroke survivor cases and their residual limitations. Select from list (multiple choice) of reasonable adjustment options. Start with ‘easy’ case before becoming more challenging with each level. Receive feedback on responses. | Employers need to be willing to accept feedback, and have time, information, and skills to complete tasks.  Employers should be committed to goals identified through task 4 (i.e., adjustments to be made). These goals should be difficult but attainable within the employer’s skill level and availability of resources. |
|  |  |  | Active learning  Goal setting | Task 2: Interactive pathway – enter in stroke survivor’s needs and get list of potential reasonable adjustments *. Information provided could suggest stakeholders who may be specially trained to support with that type of reasonable adjustment. Link to stakeholder/organisation overview.  Task 3: Go through list of potential adjustments. Identify any concerns, or where support needed (give prompts to aid reflection on health/safety, employer’s confidence/skills, and resources).  Task 4: Provide templates for reasonable adjustments passport and RTW plan (also refer back to risk assessment completed). Employer to complete/edit documents with stroke survivor and others (where consent has been given, and where applicable). Jointly identify and record realistic goals relating to reasonable adjustments and health and safety measures). Suggest minutes to be made during meetings* |  |
| 5 | PO.8. Regularly review stroke survivor’s needs (with stroke survivor) on ongoing basis as agreed (e.g., monthly basis), repeat PO. 5-7 | Knowledge, Beliefs about Consequences/ Emotion | Persuasive communication  Environmental re-evaluation | Message: Stroke survivor limitations and requirements for reasonable adjustments can change over time. This also means that health and safety measures may change over time. Provide examples from research literature, videos of stroke survivors’ real life experiences – e.g., what happened when reviews done (or not done) regularly. Re-emphasise that pre-injury level of capabilities may never completely return. | Messages should be relevant to employer, not too different from their beliefs (e.g., if they strongly believe stroke survivor’s limitations will never change). Videos need to stimulate both cognitive and affective appraisal (i.e., impact of regular reviews vs. infrequent/no reviews). |
|  |  | Skills/Beliefs about capabilities | Participation  Active learning  Public commitment  Self-monitoring of behaviour | Task 1: Contract template to complete (editable example to be provided). Enter information on role of each participant (employer/stroke survivor) in review process (give examples – e.g., employer to reflect on and maintain/obtain adequate skills for reasonable adjustments). Include what is to be covered in each review meeting (e.g., feedback, reviews of reasonable adjustments passport and risk assessment). Ask each participant to sign this document at the end to signify that they have read and understood their role in regular reviews. Additional task at end: Book in series of regular review meetings (including risk assessments also) into work calendars, set up calendar alerts.  Task 2: Following each review meeting, employer to reflect on skills/knowledge and compile list of educational/training/support needs if relevant. Suggest personal training record for employer (include template if one does not exist) to record reflection of skills and completion of training, etc. Provide link to stakeholder/organisation overview.  Suggest minutes to be made during meeting* | Employer and stroke survivor need to be motivated to do regular reviews, and be willing to publicly commit to this via the signed contract. Both parties need time, information (e.g., access to work calendars) and adequate skill to do the tasks. |
| Relevant to all steps | PO.9. Identify and liaise with appropriate stakeholders (with stroke survivor consent) or organisations, review information, or attend relevant training. | Skills/Beliefs about capabilities,  Beliefs about consequences/ Emotions,  Knowledge,  Social/Professional Role & Identity | Advance organisers | Overview of stakeholders/organisations, i.e., who to contact for support with learning about the following *  - how to communicate with the stroke survivor  - stroke in general  -employer role and responsibilities  -availability of resources  -stroke survivor’s specific capabilities/ limitations/ needs and prognosis  -impact of stroke survivor return on wider team (including carrying out risk assessments)  -identifying, organising, and reviewing adjustments that are affordable and take account of health and safety of everyone.  E.g., might include CIPD, Acas, Access to Work, trade union, NHS services, etc.*  Refer to this overview throughout toolkit, highlight in bold the stakeholders being mentioned that may be able to help at certain timepoints (and also highlight how they can help). | Schematic representations of the content or guides to what is to be learned (e.g., use of bold font and/or colour to highlight) |

*Indicates a suggestion by a workshop participant or expert advisory group member

**References**

1. Svanholm F, Turesson C, Löfgren M, Björk M. Acceptability of the eHealth Intervention Sustainable Worker Digital Support for Persons With Chronic Pain and Their Employers (SWEPPE): Questionnaire and Interview Study. JMIR human factors. 2023;10:e46878-e.

2. Greidanus MA, de Rijk AE, Frings-Dresen MHW, Tiedtke CM, Brouwers S, de Boer AGEM, et al. The Use and Perceived Usefulness of an Online Toolbox Targeted at Employers (MiLES Intervention) for Enhancing Successful Return to Work of Cancer Survivors. Journal of occupational rehabilitation. 2020.

3. Volker D, Zijlstra-Vlasveld MC, Brouwers EPM, van der Feltz-Cornelis CM. Process evaluation of a blended web-based intervention on return to work for sick-listed employees with common mental health problems in the occupational health setting. Journal of occupational rehabilitation. 2017;27(2):186-94.

4. Cadilhac DA, Andrew NE, Busingye D, Cameron J, Thrift AG, Purvis T, et al. Pilot randomised clinical trial of an eHealth, self-management support intervention (iVERVE) for stroke: feasibility assessment in survivors 12–24 months post-event. Pilot and feasibility studies. 2020;6(1):1-172.

5. Schumacher L, Armaou M, Rolf P, Sadhra S, Sutton AJ, Zarkar A, et al. Usefulness and engagement with a guided workbook intervention (WorkPlan) to support work related goals among cancer survivors. BMC psychology. 2017;5(1):34.
