# Supplementary material for: Development of a digital, self-guided return-to-work toolkit for stroke survivors and employers using intervention mapping
Source: PLOS Digit Health. 2025 Aug 6;4(8):e0000971. doi: 10.1371/journal.pdig.0000971 (PMC12327610; doi:10.1371/journal.pdig.0000971)
Supplement: S7 Table — (DOCX) [file pdig.0000971.s010.docx]

**S7. Plan for intervention design, content, scope, and sequence.**

| **Overall idea for toolkit design:** Visually stimulating (not text-heavy), interactive website, with different sections for stroke survivors and employers (and possibly a section on implementation for HR). Include questions and different pathways. Downloadable PDF documents, easy-to-find buttons.  **General ideas based on research evidence:**   - Clear overview at beginning of toolkit - Good to have downloadable PDF version (to print) in case too tiring to use electronic version [1] - Do not repeatedly ask the same questions [2] - Reminders to continue intervention [1, 2] - Toolkit needs clear structure and layout, combination of visual and textual information [3] - Scenarios should not be unrealistically positive or assumptions made about how things are/could be done in an organisation [3] - Toolkit should not be advertised (or link provided) by email only, as people have missed emails in other research [2, 3] - Potential for IT issues. E.g., IT system blocked, videos lagging, parts of intervention not visible on smaller screens [3] - Forum to consult with fellow employers (workshop participants also said this) [3]   **Who will use the toolkit?:**   - Stroke survivors who were in employment (any organisation) before their stroke, and are considering returning to work. - The stroke survivors’ line managers (to be used by HR/OH if considered useful for learning needs). To be used, regardless of post-stroke RTW experience. *Likely effective for stroke survivors that are willing to engage and have good relationship with employer (Svanholm et al., 2023)   **Access to toolkit:** Starting upon stroke survivor’s discharge from hospital. Unlimited, lifetime access for stroke survivors and employers.  **Time to complete toolkit: 5 x 15-minute modules (1.25 hours total), with additional time for suggested (optional) tasks.**  If the website had user accounts with log-in feature, their progress through the website could be saved. Progress bar could show participants their progress through the website learning (e.g., 30% complete). | | |
| --- | --- | --- |
| **Sequence, scope, and content of modules** | **Stroke survivor** | **Employer** |
| Overview | Brief introduction. State intervention goal. State that relevant stakeholders may be able to support at all stages of intervention training. Suggest that all meetings are minuted.  Provide glossary/overview of stakeholders and organisations. Include descriptions of roles and suggestions of how to contact (provide contact details if available). ***Highlight in bold stakeholder names/titles in overview and throughout toolkit.*** | |
| Step 1 | **Key focus – Readiness to start planning/preparing for RTW:**  Messages:   - Work did/may not have caused stroke and may not damage health in future. - It is good to reflect on whether ready to start planning/preparing for RTW, and to communicate this to employer. - * Include videos of stroke survivors’ success stories incorporating these approaches and actions. - Early, regular communication with employer is important (include examples - how to tell employer they have had a stroke, how to say they are ready to start RTW prep). | **Key focus – Stroke and communication:**  Messages:   - Work did/may not have caused the employee’s stroke and may not damage health in future. - Stroke can have various causes, and affects individuals differently. - Stroke survivors’ residual limitations can affect their work abilities (and can be invisible). - A sustainable return to work can be possible with the right communication and support. Rehabilitation can be a long process. Preparing and planning for work involves gradual trial-and-error approach, and action planning at regular timepoints. * Include videos of stroke survivors’ success stories incorporating these approaches and actions. - Early, regular communication with stroke survivor employees is important (include what to consider, conversation starters). - Skills and confidence for communicating with stroke survivor employees can be improved through education and training.   *Videos of employers saying how they communicated, and benefits for doing so. Show examples of good communication in videos.  *Cite relevant research and guidelines. |
| Step 2 | **Key focus: Appraisal of capabilities, limitations and needs**  Messages:   - Appraising capabilities and limitations is important for understanding what is needed for RTW. *Include video interview with stroke survivor/OT - Definition of disability according to Equality Act, definition of reasonable adjustments. - It is important to identify which needs employers should know (to provide support) | **Key focus: Employers’ roles and responsibilities**  Messages:   - Definition of disability according to Equality Act, definition of reasonable adjustments (give examples for particular limitations). - Employers have various responsibilities (include examples) in supporting the stroke survivor - Resources available to support stroke survivor may be internal or external to organisation - Reasonable adjustments are a worthwhile investment for retaining stroke survivor (better return-on-investment to retain than lose them) - Flexibility is needed (include examples of helpful strategies – e.g., dealing with short staffing).   *If stroke survivor decides to include employer in appraisal process, suggest that employer reads step 3 also before supporting with this. |
| Step 3 | **Key focus: Disclosure of needs to employer**  Messages:   - Disclosure of limitations and needs could lead to more realistic expectations and better support from employer. - Remind stroke survivors of their legal rights (e.g., Equality Act).   *Include videos/testimonials of stroke survivors discussing positive impact of disclosure | **Key focus: Understanding stroke survivors’ capabilities, limitations, and needs**  Messages:   - Reminder: Stroke can have various causes, and affects individuals differently. - Communicating with relevant stakeholders about the stroke survivor’s capabilities, limitations, and needs is important for providing tailored support. Include note on confidentiality and importance of consulting stroke survivor first.   *Videos of stroke survivors talking about specific challenges (e.g., emotional responses) |
| Step 4 | **Key focus: Planning and recording reasonable adjustments**  Messages:   - Simulation of work tasks can be helpful for further identifying stroke survivors’ capabilities, limitations, and needs (include suggestions). Some adjustments may not be realised until after RTW. - Open communication could help maintain relationship with employer. Important for decision-making and problem-solving. - Team members can be involved in preparation and planning (with stroke survivor’s consent) (e.g., workplace buddy to help with feedback and appraisal). | **Key focus: Planning and recording reasonable adjustments**  Messages:   - Simulation of work tasks can be helpful for further identifying stroke survivors’ capabilities, limitations, and needs (include suggestions). Some adjustments may not be realised until after RTW. - Employers may need help in carrying out/providing reasonable adjustments and other RTW process actions if needed (e.g., RTW plan). Should offer all possible adjustments before making decision about stroke survivor continuing in role. - It is important to think about how stroke survivor’s return may impact wider team. Include tips for discussion, e.g., example phrases, video showing employer carrying out discussions in way that respects stroke survivor’s wishes regarding disclosure. - Team members can be involved in preparation and planning (with stroke survivor’s consent) (e.g., workplace buddy to help with feedback and appraisal).   *Videos/written narratives showing benefits when employers liaised with stroke survivors and other stakeholders (e.g., occupational therapists) about stroke survivors’ abilities. |
| Step 5 | **Key focus: Ongoing review of capabilities, limitations, needs, and reasonable adjustments**  Messages:   - Capabilities, limitations, and needs can change during recovery from stroke; recovery can be long-term and pre-injury level of capabilities not always reached. Cite research evidence. - It is important to meet regularly with employer to review needs. | **Key focus: Ongoing review of capabilities, limitations, needs, and reasonable adjustments**  Messages:   - Stroke survivor’s capabilities, limitations, and needs can change during recovery from stroke; recovery can be long-term and pre-injury level of capabilities not always reached. Cite research evidence. - To ensure continuation of adequate (tailored) support, it is important to meet regularly with stroke survivor to review needs. - Resources, health and safety measures, and confidence/skills for making adjustments can also change.   *Videos of stroke survivor’s RTW experiences, what happened when regular reviews done (or not done). |
| Delivery of toolkit | - Website/e-learning online, accessible from any desktop, tablet or phone device - User accounts, with log-in to access toolkit | |
| Resources needed | Funding needed for the following:   - Initial development and set-up of toolkit, guidance from expert? - Payment for those involved in producing videos for toolkit (e.g., stroke survivors, employers, technical staff) - Payment for stroke survivors sharing real life experiences (e.g., promotional events to advertise toolkit?) - Ongoing maintenance of evidence-based/legal content and technical support | |
| Materials needed | - Platform to use for coalition of people from different organisations to support use of toolkit - Users to have access to Internet and desktop, tablet or phone device - Protected time to progress through website | |

**References**

1. Svanholm F, Turesson C, Löfgren M, Björk M. Acceptability of the eHealth Intervention Sustainable Worker Digital Support for Persons With Chronic Pain and Their Employers (SWEPPE): Questionnaire and Interview Study. JMIR human factors. 2023;10:e46878-e.

2. Volker D, Zijlstra-Vlasveld MC, Brouwers EPM, van der Feltz-Cornelis CM. Process evaluation of a blended web-based intervention on return to work for sick-listed employees with common mental health problems in the occupational health setting. Journal of occupational rehabilitation. 2017;27(2):186-94.

3. Greidanus MA, de Rijk AE, Frings-Dresen MHW, Tiedtke CM, Brouwers S, de Boer AGEM, et al. The Use and Perceived Usefulness of an Online Toolbox Targeted at Employers (MiLES Intervention) for Enhancing Successful Return to Work of Cancer Survivors. Journal of occupational rehabilitation. 2020.
