# Supplementary material for: Development of a digital, self-guided return-to-work toolkit for stroke survivors and employers using intervention mapping
Source: PLOS Digit Health. 2025 Aug 6;4(8):e0000971. doi: 10.1371/journal.pdig.0000971 (PMC12327610; doi:10.1371/journal.pdig.0000971)
Supplement: S8 Table — (DOCX) [file pdig.0000971.s011.docx]

**S8. Summaries of pre-test feedback.**

Feedback provided by:

- Stroke survivors (n=7)
- Employers (managers n=2, small business owners n=2)
- Healthcare professionals (HCP) (n=3)
- Trade union representative (TUR) (n=1)

TTEAM=Toolkit for Transitioning to Employment After stroke through Mutual support

**Table 1.** Theme summaries for acceptability, ease of use/learnability, accessibility, inclusivity, perceived usefulness, and issues affecting use.

| **THEME/CODE** | **SUMMARY** |
| --- | --- |
| *Note: Over half of the stroke survivors looked at both versions. One said they would want to see what content the employer was being provided with.* | |
| **ACCEPTABILITY/WILLINGNESS TO USE (TAM)** | *Stroke survivors, employers and HCPs believed TTEAM would fill a gap in return-to-work resources. Some expressed a desire to use it themselves, and a potential use for broader application was stated.* |
| **ACCESSIBILITY & INCLUSIVITY (ICF)** | |
| *Features they did NOT like* | *One stroke survivor with aphasia expressed dislike for TTEAM's pop-up descriptions. A stroke survivor and stroke survivor/employer thought the text-dense slides (at the beginning) and overall length may be overwhelming for those with cognitive impairments and/or fatigue.* |
| *Features they liked* | *Stroke survivors found TTEAM content clear, concise, and easy to read. An HCP with Attention Deficit Hyperactivity Disorder appreciated the multiple information modalities.* |
| **PERCEIVED EASE OF USE & LEARNABILITY (TAM & SUS)** | |
| *Features they did NOT like* | *A stroke survivor and an employer experienced confusion regarding the menu of contents. Employers also reported issues with PDF access and location of areas on a body map diagram. One HCP mentioned excessive text density on one slide (employer version). Stroke survivors highlighted difficulties with saving progress, navigating back from external links, and time constraints for employers completing tasks.* |
| *Features they liked* | *Stroke survivors and employers praised TTEAM for its clear navigation, interactivity, and flexible, step-by-step structure. They appreciated the clear instructions, progress indicators, and mix of activities. One HCP reported smooth functionality with Microsoft Edge, slide format, and ability to complete it in stages.* ***Note: Across themes: HCP and employers liked mix of interactive elements in employer version (for sustaining attention and engagement).*** |
| **PERCEIVED USEFULNESS (TAM)** | |
| *Useful TTEAM content* | *TTEAM was praised by stroke survivors, employers, and HCPs for its empowering educational content, personal stories/videos, and practical tools (e.g.,. job demands analysis, reasonable adjustments passport return-to-work plan).  Employers thought TTEAM would be useful for all sizes of organisations. The tools were valued for encouraging open communication between stroke survivors and employers. Stroke survivors and employers also believed content regarding the wider team (employer version) and stroke survivors' emotional symptoms/needs was very important and useful.   Overall, TTEAM was described by all as containing key information, very useful.* |
| **TECHNICAL ISSUES (ICF)** | *Technical issues included videos loading slowly or their sound not working, the inability to read PDFs with a narrator-speech tool, and incompatability of Xerte with the Safari Internet browser.* |
| **USER SATISFACTION (SUS)** | |
| *Things they liked* | *Stroke survivors and employers described TTEAM as a massive leap forward compared to other available return-to-work guidance. They found TTEAM comprehensive, and saw it as a good starting point for line managers and stroke survivors. One employer believed it would help translate evidence into practice, while a stroke survivor found it encouraging, reassuring, and empowering.* ***Note: Across themes: TTEAM seen as filling a gap, all key information, more comprehensive than anything else currently available. Content is empowering for users, and takes into account emotional impact of stroke.*** |
| *Things they did NOT like* | *One stroke survivor expressed distrust related to appearance of a human resources manager/stroke survivor in a video about stroke recovery. Employers did not like use of the term, "stakeholders." One employer thought there needed to be re-consideration of the balance/valence of content at the start, without causing unrealistic employer expectations.* |
| **SUGGESTIONS FOR IMPROVEMENT** | |
| **1. To improve ease of use and learnability with TTEAM** | |
| *Instructions* | *To improve ease of use/learnability in both versions, suggested improvements related to the initial slides (i.e., instructions, inclusion of an overview of the TTEAM process), user learning styles, the menu of contents, PDF access, and saving of progress.* |
| *Features to add/change to meet all user learning styles* |  |
| *Menu of contents* |  |
| *Saving progress* |  |
| *Accessing PDFs* |  |
| *Overview of TTEAM* |  |
| **2. To improve accessibility and inclusivity of TTEAM** | |
| *Disability-related user strategies* | *To improve accessibility and inclusivity of TTEAM, suggested improvements included audio versions of TTEAM, and inclusion of instructions for people with disabilities, e.g., look at TTEAM when concentration is best.* |
| **3. To improve usefulness of TTEAM** | |
| *Guidance on communication* | *To improve usefulness of TTEAM, suggested improvements included additional conversation aids for employers and stroke survivors, scenario-based questions to aid employer reflection, a body map tool to aid identification of reasonable adjustments, additional signposting and videos/personal stories, a stage-based overview of the return-to-work process, and minor changes to facilitate user engagement, and improve accuracy/relevancy of content. Employers and HCPs thought TTEAM should remain as it is (i.e., not condensed into a 'light' version).  Note: Stroke survivor comment re. additional conversation aids goes against employers' views (they were happy with the content regarding communication). This stroke survivor had a human resources background, and also suggested a checklist of employers' responsibilities at each stage, and employers did not agree with this either.* |
| *Other additional content to enhance usefulness* |  |
| *Adapted versions of TTEAM* |  |
| *Accuracy/relevance of content* |  |
| *Changes to improve user experience/engagement* |  |
| **4. Technical issues** | |
| *Internet browser compatibility* | *One stroke survivor suggested improving Internet browser compatibility or instruction regarding their use.* |

**Table 2 included on following page.**

**Table 2.** Summary of feedback regarding how to increase awareness of- and provide access to TTEAM.

| **SUGGESTION** | **SUMMARY** |
| --- | --- |
| **Signposting via National Health Service, charities, employers, and other organisations** | *Stroke survivors, the TUR, and employers suggested people should be made aware of TTEAM via charity (e.g., Stroke Association, Same You, Different Strokes, Attend ABI) websites, e.g., included in resources, and social media. They also suggested the Department for Work & Pensions and HCPs could support its promotion, e.g., General Practitioners, vocational rehabilitation practitioners/therapists, local rehabilitation centres, integrated stroke delivery networks.*  *A small business owner suggested it could be empowering for stroke survivors if the National Health Service gave them access to TTEAM, and then they shared it with their employer. They also believed this could equip HCPs who may not be confident in talking to stroke survivors about work. Employers also suggested stroke survivors be presented with information about TTEAM using multiple channels at various points/levels along their recovery journey, e.g., inpatient therapy session, General Practitioner, charities, their employers, occupational health providers, human resources.*  *(TUR suggestion) Employer should also forward it to stroke survivor as soon as they are aware stroke has happened. Suggested TTEAM should also be made available on employers' intranet, occupational health provider websites, Trade Union Congress website.*  *HCPs suggested raising awareness/promoting TTEAM via local employment teams and social prescribers in councils, private occupational therapists, occupational psychologists, the Royal College of Occupational Therapists and vocational rehabilitation networks, voluntary organisations like the Disability Law Service or Mindful Employer, Job Centre Plus, the Department for Work & Pensions, Acas, Access to work (within recommendation reports), well-being departments of organisations, National Health Service stroke teams, charities, occupational health providers, and the Chartered Institute for Personnel Development website. HCPs also suggested sending TTEAM to National Health Service occupational therapy departments who provide vocational rehabilitation. They have long waiting lists and time constraints in rehabilitation provision, and could signpost to TTEAM (it could provide return-to-work guidance where they are not available).* |
| **Activities and resources to raise awareness/promote TTEAM** | *Stroke survivors suggested a network (e.g., like 'Reconnect') where users can access peer mentoring and support in the use and application of TTEAM. Mentors may be other employees in the same organisation, or outside of organisation. One stroke survivor suggested asking people that sign up, if they mind their profile and contact details being shared with other members of network. Stroke survivors suggested people who agree to provide support could be approached about going into organisations to talk about their experiences returning to work post-stroke, and TTEAM.*  *(Re. TTEAM promotion) One employer said TTEAM could be advertised on recognised awareness days (e.g., International Stroke Day), e.g., by connecting with OT firms that support industry, such as Optima Health and also human resources within the organisation. Another employer agreed with aligning promotion with recognised awareness days, and thought having stroke survivors go into organisations to talk about their experiences on these days would make it more personable, impactful, and memorable. This person thought stroke survivors could be sourced for this through charities, and recommended having a designated person to book them in advance (e.g., six months ahead of time).*    *A senior manager wondered if the logistics behind the above could be challenging. Suggested a promotional video may be just as effective as having stroke survivors present at awareness day events.*  *A small business owner suggested holding events for organisations and individuals interested in using TTEAM. An opportunity for those people to gather and obtain information they need, and feel heard and understood (employers as well as stroke survivors).* |
