# Supplementary material for: Development of a digital, self-guided return-to-work toolkit for stroke survivors and employers using intervention mapping
Source: PLOS Digit Health. 2025 Aug 6;4(8):e0000971. doi: 10.1371/journal.pdig.0000971 (PMC12327610; doi:10.1371/journal.pdig.0000971)
Supplement: Attachment — Submitted filename: Peer reviewer responses_PDIG-D-25-00219.docx [file pdig.0000971.s013.docx]

**Manuscript title: Use of intervention-mapping to develop a self-guided, digital return-to-work toolkit for stroke survivors and employers**

**Reference number: PDIG-D-25-00219**

Dear all,

Thank you for the time and effort you have invested in reviewing our manuscript. We appreciate the constructive feedback, which has helped us improve the quality of our work. Below, we provide a detailed response to each comment, with corresponding revisions made in the manuscript. For clarity, we have structured our responses as follows:

- Reviewer comments are shown in bold.
- Our responses are in regular text, prefaced by "Response." Direct quotes from the manuscript are in italic font.
- Changes in the manuscript are shown as tracked changes. We have also attached a clean version without tracked changes shown.

Reviewer 1

1**. [Methods]** **Clarify how workshop and advisory group participants were recruited (e.g., sampling strategy, inclusion/exclusion criteria).**
Response: Thank you for your comment. We can confirm that details on the recruitment of the advisory group members and workshop participants are already present in the text. For clarity, we present the direct quotes from the manuscript below:

*“In July 2022 KC identified potential members via convenience sampling, and invited them to attend three advisory group meetings over two years, to advise on data collection (relating to the needs assessment and workshops), a ‘logic model of change,’ and pretesting of TTEAM prototypes. Potential members were selected based on their clinical, professional, and/or academic expertise, or lived experience of stroke, VR, implementation science, human resources (HR), or occupational health (OH). Informed consent was not required because their role involved advising on the project and overseeing progress, rather than contributing personal data” (lines 209-216).*

*“Employers were recruited through convenience and snowball sampling. The study was advertised February 2023-January 2024, through social media, gatekeepers, and business networking events in the Midlands, UK. Eligible participants were aged 18 years or older; worked in an occupational role involving staff responsibility; and proficient in use of English language” (lines 243-247).*

2. [**Methods] Justify sample sizes for workshops (n=12 employers) and pretesting (n=15) with power calculations or saturation references.**
Response: As this study followed the intervention mapping approach, no formal sample size calculations were undertaken for the workshops (n=12) or pretesting (n=15). Intervention mapping does not prescribe specific sample sizes for stakeholder engagement; rather, it emphasizes the iterative involvement of diverse stakeholders across stages of intervention development. During this study, sample sizes were determined pragmatically based on stakeholder availability and engagement, ensuring input from a diverse range of relevant perspectives (e.g., employers, stroke survivors, healthcare professionals, and experts). We have clarified this point in the manuscript, as follows: *“Sample sizes for the workshops were determined pragmatically, based on participant availability and engagement in the co-design process, in line with the iterative and participatory nature of the IM approach [55]” (lines 248-250).*

3. [**Abstract - Methods] Provide specific examples of "theory-based pretesting" methods used (e.g., cognitive walkthroughs, usability metrics).**

Response: We have added further detail into the methods section of the abstract to show that the pretesting involved task-based usability testing and advisory group discussions. To keep within the 300 word limit, we removed reference to the testers as being members of the project’s advisory group (this information has been retained within the main body of the manuscript). The revised text now reads: *Theory-based pretesting (task-based usability review, advisory group discussions) was conducted with employers (n=4), stroke survivors (n=7), and healthcare professionals (n=4)” (lines 34-36).*

To improve clarity, we have also included a brief summary of tasks that individuals completed as part of the task-based usability review exercise. This has been included in the methods section: *“During this time, they were asked to complete a series of guided tasks simulating typical use, such as adjusting accessibility settings, navigating pages, viewing content in various formats (text, video, PDF), and exploring the prototype(s) more broadly” (lines 222-225).*

4. [**Results]** **Include quantitative usability metrics (e.g., System Usability Scale scores) alongside qualitative feedback to strengthen pretesting claims.**

Response: The System Usability Scale (SUS) was not administered as a quantitative measure during this study; therefore we are unable to report usability metrics. SUS constructs, along with those from the Technology Acceptance Model and the International Classification of Functioning, Disability and Health, were used to guide construction of open-ended feedback questions and the framework analysis. As the study was exploratory with a small sample (n=15), we focused on gathering in-depth qualitative insights to inform future development of the TTEAM intervention. We recognise the value of taking a mixed methods approach in future pretesting of TTEAM and in a feasibility study, and have suggested this in the discussion section, as follows:

*“Refinement and more formal pretesting of TTEAM with larger, diverse user groups is needed.”* (lines 583-584)

*“Observing stroke survivors’ interactions with TTEAM may identify any remaining accessibility or usability issues. Quantitative usability metrics, e.g., System Usability Scale scores, and qualitative data from focus groups could enhance the validity of findings, inform decisions regarding the stroke survivor target audience, and facilitate exploration of solutions to any persistent barriers.”* (lines 589-593)

*“A detailed plan for a feasibility study with an embedded, mixed methods effectiveness-implementation hybrid design, aligned with IM step 6 and Medical Research Council guidance [104, 105], is also required.”* (lines 616-619

5. **[Results] Show comparative data on how this toolkit improves upon existing resources through tables or direct comparisons.**

Response: Thank you for this suggestion. As this was an intervention development study guided by the Intervention Mapping approach (Steps 1–4), the focus was on co-designing and pretesting the prototype rather than evaluating comparative effectiveness. In line with standard practice for digital health intervention development, formal evaluation (e.g., collection of comparative data across different interventions) was not conducted. As such, we are unable to present comparative data on TTEAM and other return-to-work toolkits or guidance at this stage.

6. **[Ethical compliance]** **Explicitly state ethics approval details (institution, reference number) in the Methods section.**

Response: We have moved the ethical approval details from the end of the manuscript to the methods section. It reads as follows: *“Ethical approval was obtained from the University of Nottingham Faculty of Medicine & Health Sciences Research Ethics Committee (ref: FMHS 166-1122). Formal, informed consent was obtained verbally from employer workshop participants” (lines 180-182).*

7. **[Toolkit accessibility] Address potential digital literacy barriers: 16% of UK stroke survivors have post-stroke cognitive impairments affecting technology use.**

Response: Thank you for this suggestion. We acknowledge the importance of this, and have suggested involving stroke survivors with a range of cognitive impairments (and severities of these impairments) to pre-test TTEAM a second time, following initial refinements. We have also specifically acknowledged digital literacy as a barrier, alongside other barriers such as limited access to technology, cultural beliefs regarding stroke recovery, and lack of perceived benefit for engaging with digital interventions. Based on these highlighted barriers, we have suggested involving stroke survivors (and employers) from a range of socio-economic and cultural backgrounds, occupational industries, and geographical locations in future pretesting and evaluation of TTEAM. Reference to equality, diversity and inclusion guidance is suggested, as well as exploration of the usefulness of an embedded chatbot tool and/or paper versions of TTEAM. This additional text is all in the discussion section, as shown below:

*“Refinement and more formal pretesting of TTEAM with larger, diverse user groups is needed. In this study, issues included PDF and narrator-speech tool incompatibility, text density, unclear navigation instructions, and lack of function to save progress. Furthermore, up to 60% of stroke survivors experience cognitive impairment post-stroke [99] which may affect their ability to recall login details, follow complex instructions, fill out forms, etc. Following initial refinements, further pretesting should include stroke survivors with cognitive impairments of varying types and severities. Observing stroke survivors’ interactions with TTEAM may identify any remaining accessibility or usability issues. Qualitative data from focus groups and quantitative usability metrics, e.g., System Usability Scale scores, could enhance the validity of findings, inform decisions regarding the stroke survivor target audience, and facilitate exploration of solutions to any persistent barriers. For example, some users may prefer a printable PDF version of TTEAM, or guided support (e.g., via an embedded chatbot tool). Paper versions of TTEAM may alter the application of behaviour change methods, and diminish the potential effectiveness of TTEAM. Therefore, if implemented, these alternatives should be evaluated separately from the digital versions as comparators.”* (lines 583-598)

*“Research also indicates that stroke survivors can lack digital literacy, have limited access to technology due to economic instability or rural locations, or lack perceived benefit for engaging with digital interventions [100]. These issues may be especially prevalent among those working in industries with little need for digital skills, like unskilled manual labourers. To avoid excluding these potentially underserved populations, stroke survivor and employer representatives from various socio-economic backgrounds, occupational industries, and geographical locations should be recruited for pretesting, and during future evaluation of TTEAM. Language, cultural, religious, and ethnic differences may also hinder stroke survivors’ engagement with self-management interventions. For instance, where ethnic minority communities believe only medication and rest are required for complete stroke recovery [101]. To optimise accessibility and inclusion for underserved groups, setup of the pretesting group and planning of TTEAM’s implementation and evaluation (IM steps 5-6) should align with relevant equality, diversity, and inclusion guidance [102, 103].” (lines 599-611)*

8. **[Toolkit accessibility] Propose alternative formats (e.g., print guides) for users with accessibility needs.**

Response: Whilst our stakeholders strongly suggested that TTEAM be digital and interactive, and accessibility is optimised within Xerte (e.g., accessible fonts and font sizes, customisable themes), we acknowledge that some stroke survivors may not have access to assistive technology, such as screen readers, to support prolonged use of a screen. We also acknowledge that a paper version may support use where a stroke survivor/employer may not have ongoing access to a computer or tablet. At the same time, the digital elements, e.g., videos, represent key applications of the behaviour change methods within TTEAM (both the stroke survivor and employer versions), and so their omission may dilute its potential effectiveness. We have suggested exploring the usefulness of downloadable, printable PDF versions of TTEAM during the next round of pretesting. It may be possible to implement paper versions as comparators within a feasibility study. The revised text in the discussion section reads as follows:

*“For example, some users may prefer a printable PDF version of TTEAM, or guided support (e.g., via an embedded chatbot tool). Paper versions of TTEAM may alter the application of behaviour change methods, and diminish the potential effectiveness of TTEAM. Therefore, if implemented, these alternatives should be evaluated separately from the digital versions as comparators.” (lines 593-598)*

9. **[Abstract] Specify the platform used (Xerte) and its accessibility features.**

Response: Thank you for this suggestion. Xerte had already been specified as the platform within the abstract, however examples of its accessibility features have been added, as follows: *“It exists as two Xerte eLearning packages, with accessibility features such as screen reader compatibility and keyboard navigation” (lines 41-42).*

This revision led to an excessive number of words in the abstract (word count: 300), so we have removed mention of exploration of its scalability, and removed the word, “uniquely,” in front of “comprehensive” on line 48.

10. [**Non-Technical Summary] Replace subjective claims like "response was very positive" with concrete metrics (e.g., "X% of users reported improved confidence").**

Response: We have removed the phrase, “response was very positive” from the Non-Technical Summary (now re-named Author Summary and edited, in line with the Editor’s comment and PLOS Digital guidance). As this was a qualitative intervention development study with a small, purposive sample (n=15), we did not collect quantitative data on outcomes such as confidence. All findings have been reported based on framework analysis (used to structure consultative feedback from advisory group members), and are presented without subjective generalisations. To enhance credibility of the feedback summary, the first author discussed coding decisions and interpretations with the research team. This has now been added to the methods section: ***“****KC (first author) had experience of framework analysis and independently carried out the process. Coding decisions and interpretations were discussed with the research team to enhance credibility of the feedback summary*.*” (lines 234-236).*

11. **[Data Availability] Current statement meets requirements but should clarify timelines for data access.**

Response: Thank you for this suggestion. We have also noted the comment from the Editor regarding an exemption request for not all data being made publicly available. We have updated the data availability statement to clarify that anonymised workshop transcripts may be shared with other researchers following publication of this article, subject to research ethics approval and a data-sharing agreement. The reason for this restriction is because, in the verbal consent form for workshop participants, all agreed to the following statement: *“I agree that my anonymous research data will be stored and used to support other research in the future, and shared with other researchers including those working outside the University.”*

The revised text in the manuscript reads: “*Access to anonymised workshop transcripts is restricted due to the potential inclusion of identifiable and confidential participant information. This data management approach was approved by the University of Nottingham Faculty of Medicine & Health Sciences Research Ethics Committee (Ref: FMHS 166-1122). Participants consented to the use of anonymised quotes in publications and to the sharing of anonymised data with other researchers. Full workshop transcripts may be shared upon request with qualified researchers for research purposes, following publication, and subject to research ethics approval and a data-sharing agreement. Requests should be directed to the data custodian, Professor Kathryn Radford (*[*mczkar1@exmail.nottingham.ac.uk*](https://mczkar1@exmail.nottingham.ac.uk/)*), or to the Research Ethics Committee at fmhs-researchethics@nottingham.ac.uk. Other study materials, including workshop PowerPoint slides, can be found at:* [*http://doi.org/10.17639/nott.7520*](http://doi.org/10.17639/nott.7520)*”*

12. **[Financial Disclosure] Complete but should explicitly state funders' roles in study design/implementation.**

Response: The funders had no role in study design or implementation. The following sentence has been added to the Funding section: *“The funders had no role in study design, data collection and analysis, decision to publish, or preparation of the manuscript.” (lines 665-667).*

Reviewer 2

**Overarching comment: My only major concern is that in its present form, the study describes a development study. This is fine as I find the quality and thoroughness of presentation here to be high, however, the developmental aspect could be made more explicit as readers not familiar with all the steps of intervention-mapping may misinterpret the scope of the study and expect outcomes data. The development aspect could be made more clearer in the title, abstract, and in key paragraphs throughout the paper (e.g. last introduction paragraph and first Discussion paragraph).**

Response: Thank you for this helpful suggestion. We have made changes throughout the manuscript to reflect this, as outlined below:

Title: ‘Development’ now appears at the start to emphasise this aspect of the study. The title now appears as: *“Development of a digital, self-guided return-to-work toolkit for stroke survivors and employers using intervention mapping.”* (lines 1-2)

Abstract: The final sentence in the background section has been altered. Previously it stated that the study involved co-design of a toolkit, but this has been altered to ‘developed’, as follows: *“This study developed a self-guided return-to-work toolkit for stroke survivors and employers.”* (line 29)

We have also made changes to highlight that formal testing was not conducted, instead a ‘pre-test’ was conducted via an informal prototype review with the advisory group. The group provided feedback on TTEAM in a consultative capacity and were not research participants. The revised text now reads:

*“Theory-based pretesting (task-based usability review, advisory group discussions) was shaped by prototype review with advisory group members, including employers (n=4), stroke survivors (n=7), and healthcare professionals (n=4). Framework analysis was used to structure feedback related to acceptability, ease of use/learnability, accessibility, inclusivity, perceived usefulness, and technical or environmental issues; no personal data were analysed*” (lines 34-39).

Last introduction paragraph: The order of words in the first sentence have been changed to emphasise the developmental aspect of the study: *“This study aimed to develop a digital, self-guided RTW toolkit for stroke survivors and employers using the intervention mapping (IM) approach”* (lines 163-164)

Methods section: We have now added text to emphasise that intervention mapping steps 1-4 focus on intervention *development.* The text now reads: *“In this study, IM steps 1-4 were followed to develop TTEAM, with plans to complete refinement and steps 5-6 in future work”* (lines 187-188)

We have also added/edited text to emphasise the role of the advisory group in their consultative capacity, rather than as formal testers of the toolkit:

*“For example, during the ‘pre-testing’ of TTEAM (IM step 4), they were invited to provide expert and practical feedback on prototypes in a consultative capacity, and not as research participants. Although some informally disclosed their stroke-related impairments to KC (first author), no personal data were collected.”* (lines 216-220)

*“Framework analysis [61] was used to structure consultative feedback from advisory group meeting transcripts and email responses, guided by the question constructs.”* (lines 231-233)

First discussion paragraph: the word, ‘development’ has been added in: *“In this development study, use of the IM approach supported the application of theory, evidence, and stakeholder input to ensure TTEAM effectively meets users’ needs when planning and managing RTW post-stroke”* (lines 647-649)

1. [**Abstract] Please define acronym RTW.**
Response: Thank you for pointing this out. Upon examination of the abstract, we did not feel an acronym was necessary, so have removed it and replaced it with *“return-to-work”* *(line 41).*

2. [**Abstract] Readers not familiar with intervention-mapping approach (steps 1-4), may not understand there are subsequent steps; could this be made more clear?**

Response: Thank you. We have clarified that the intervention-mapping approach consists of six steps, and that this study focused on steps 1–4. The revised text now reads: *“Steps 1-4 of the six-step intervention-mapping approach were followed (line 30).”*

3. **[Introduction] The Introduction/background is quite long, and could be focused better, in a manner to motivate the need and rationale behind the study more forcefully. It was hard to maintain interest in reading it due to its length and wordiness. I would suggest removing tangential passages and/or making the following passages more concise:**
*“Authors suggested one of these interventions was effective because it encouraged women recovering from gynaecological surgery to engage in their recovery [49].”
"Stroke survivors and employers may access private VR support through their organisations (via employee assistance programs, OH provision, or group income protection insurance, for example) , or through UK government schemes such as Access to Work, or the third sector. However, support offered may fall short of meeting their needs, because it is not stroke-specific nor part of a comprehensive rehabilitative program [20]."
"In a UK survey in 2018 (N=11,134), 37% of working-age stroke survivors stopped working following stroke [27]. Specific reasons for this were not directly cited. Nevertheless, the economic, health, and social costs associated with stroke survivors’ loss of employment are vast, impacting stroke survivors, employers, and society. Reduction or loss of employment among UK stroke survivors cost £1.6 billion in 2015, and is predicted to rise 136% by 2035 [28]. Improving RTW support for the long-term sick and disabled is high priority on the UK Government’s agenda [29, 30]."*

Response: Thank you for this suggestion. We appreciate the guidance in making the introduction more concise. The suggested passages have been shortened, and edits made throughout the introduction section to improve flow and readability (please see tracked changes throughout the section). In total, we have reduced the length by approximately 200 words.

4. **[Methods] Sampling: Please provide fuller demographics for participants (age, sex, time-since-stroke, etc…).**

Response: During steps 2–4 of the intervention mapping process, we collected only demographic data directly relevant to the aims of intervention development - specifically, stroke survivor status, occupational role, organisational setting, and workshop attendance. The purpose of this was to map diversity of perspectives, understand organisational contexts, and demonstrate stakeholder involvement throughout the intervention development process. In line with the UK Data Protection Act 2018 and the principle of data minimisation, we did not collect additional demographic data that were not needed at this stage. We agree that fuller demographic information (e.g. age, sex, time-since-stroke) would be valuable in future testing and implementation phases, and we will incorporate this into our next stage of evaluation.

5. **[Methods] Framework analysis: specify coder training and any intercoder reliability process.** Additional text has been added, explaining that the first author had previous experience of this analysis method, and was responsible for conducting the analysis (with coding and interpretations checked with the research team). The revised text now reads, ***“****KC (first author) had experience of framework analysis and independently carried out the process. Coding decisions and interpretations were discussed with the research team to enhance credibility of the feedback summary”* *(lines 234-236).*

6. **[Methods] Figure 2 may be better suited for Supplemental Information, with each screenshot parsed out into separate figures because it is relatively dense with small font in current format.**

Response: We feel unable to put Figure 2 in the supporting information. It is a key diagram illustrating the proposed theory behind how the toolkit will work in practice. Removing Figure 2 would also affect Table 4, because it defines the behaviour change methods and underlying theories integrated within the toolkit, and maps them directly to the behaviour objectives defined in Figure 2. We appreciate your comment and have reduced the text within Figure 2 to make it less dense. We hope this addresses your concern, but welcome any further feedback you may have.

7. **[Discussion] It would be helpful to provide further details re: the planned feasibility study design (primary feasibility metrics, progression criteria, etc…).**

Response: Thank you for this suggestion. We have added in details regarding a future hybrid study design, feasibility metrics, progression criteria, and outcomes, and who may be involved in helping to develop the plans for this study. The additional text is shown below:

*“Future plans should also be informed by consultation with a core advisory group, including stroke survivors, employer and healthcare stakeholders, and professionals with expertise in implementation science, digital learning, health economics, health and safety, and equality, diversity, and inclusion. For example, further work is needed to establish who needs to do what and when to facilitate successful implementation (IM step 5). A detailed plan for a feasibility study with an embedded, mixed methods effectiveness-implementation hybrid design, aligned with IM step 6 and Medical Research Council guidance [104, 105], is also required. This study should evaluate key feasibility outcomes such as recruitment and retention rates, acceptability, usability, intervention adherence (e.g., completion of TTEAM sections, or ‘steps’), and completion of outcome measures. Progression criteria will be defined a priori, informed by literature on similar interventions and advisory group input, to determine whether a definitive trial is warranted (e.g., >70% recruitment and retention, high acceptability and usability ratings).” (lines 612-624)*

*“Potential outcomes include the TTEAM change or performance objectives such as employers’ confidence or competence in making reasonable adjustments, behavioural outcomes such as employers providing reasonable adjustments in a timely way, as well as broader impacts on health and quality-of-life improvement, and social and economic factors, including cost-effectiveness. The use of mixed methods will also enable exploration of TTEAM’s change mechanisms (i.e., the theory behind how and why it brings about intended outcomes), evaluation of its implementation and the strategies employed, and whether any refinements are needed” (lines 625-632)*

8. **[Discussion] It would be helpful to discuss generalizability outside the UK, especially differing employment and disability legislation.**

Response: Thank you for this suggestion. Given that our systematic review (part 1 of our needs assessment) was international, a lot of the data that informed our identification of users’ needs had been collected in countries outside of the UK, including the United States, South Africa, New Zealand, Sweden, Canada, Barbados, Denmark, the Netherlands, and Australia. The reference for this review is below:

Craven K, De B, Holmes J, Fisher R, Radford KA. Factors influencing employers’ support for employees with acquired brain injuries or mental illness to return to- and stay in work: A qualitative systematic review. *Work.* 2024 Jan 12;1–29.

Overall, the needs assessment findings influenced the priority areas of focus within TTEAM and the content included. We feel the majority of content is transferable to other countries, aside from the content that specifically relates to UK employment and disability legislation. We have added in text within the discussion as follows:

*“Although TTEAM was developed in the UK, much of its content, particularly guidance on communication, RTW planning, and monitoring of adjustments, is likely to be transferable to settings in other countries, seeking to implement TTEAM. However, content related to UK-specific employment and disability legislation would need to be adapted to align with their national legal and policy frameworks.” (lines 638-642)*

We hope that our revisions address all concerns raised. Please let us know if any additional changes are required.

Thank you again for your time and constructive feedback.

Sincerely,
Kristelle Craven
On behalf of all co-authors
